# Comprehensive *In Silico* Analysis of RNA Silencing-Related Genes and Their Regulatory Elements in Wheat (*Triticum aestivum* L.)

**DOI:** 10.1155/2022/4955209

**Published:** 2022-09-19

**Authors:** Zobaer Akond, Hafizur Rahman, Md. Asif Ahsan, Md. Parvez Mosharaf, Munirul Alam, Md. Nurul Haque Mollah

**Affiliations:** ^1^Bioinformatics Lab, Department of Statistics, University of Rajshahi, Rajshahi, Bangladesh; ^2^Agricultural Statistics and Information & Communication Technology Division, Bangladesh Agricultural Research Institute, Gazipur, Bangladesh; ^3^Institute of Environmental Science, University of Rajshahi, Rajshahi, Bangladesh; ^4^Department of Microbiology, Rajshahi Institute of Biosciences, University of Rajshahi, Rajshahi, Bangladesh; ^5^School of Business, Faculty of Business, Education Law and Arts, University of Southern Queensland, Toowoomba, QLD 4350, Australia; ^6^Molecular Ecology and Metagenomic Laboratory, Infectious Diseases Division, International Centre for Diarrheal Disease Research, Bangladesh (icddrb), Bangladesh

## Abstract

Dicer-like (DCL), Argonaute (AGO), and RNA-dependent RNA polymerase (RDR) are known as the three major gene families that act as the critical components of RNA interference or silencing mechanisms through the noncoding small RNA molecules (miRNA and siRNA) to regulate the expressions of protein-coding genes in eukaryotic organisms. However, most of their characteristics including structures, chromosomal location, subcellular locations, regulatory elements, and gene networking were not rigorously studied. Our analysis identified 7 *TaDCL*, 39 *TaAGO*, and 16 *TaRDR* genes as RNA interference (RNAi) genes from the wheat genome. Phylogenetic analysis of predicted RNAi proteins with the RNAi proteins of *Arabidopsis* and rice showed that the predicted proteins of TaDCL, TaAGO, and TaRDR groups are clustered into four, eight, and four subgroups, respectively. Domain, 3D protein structure, motif, and exon-intron structure analyses showed that these proteins conserve identical characteristics within groups and maintain differences between groups. The nonsynonymous/synonymous mutation ratio (Ka/Ks) < 1 suggested that these protein sequences conserve some purifying functions. RNAi genes networking with TFs revealed that ERF, MIKC-MADS, C2H2, BBR-BPC, MYB, and Dof are the key transcriptional regulators of the predicted RNAi-related genes. The *cis*-regulatory element (CREs) analysis detected some important CREs of RNAi genes that are significantly associated with light, stress, and hormone responses. Expression analysis based on an online database exhibited that almost all of the predicted RNAi genes are expressed in different tissues and organs. A case-control study from the gene expression level showed that some RNAi genes significantly responded to the drought and heat stresses. Overall results would therefore provide an excellent basis for in-depth molecular investigation of these genes and their regulatory elements for wheat crop improvement against different stressors.

## 1. Introduction

Plants by nature establish some specific molecular mechanisms to survive in diverse conditions in their life span. Gene/RNA silencing also called RNA interference (RNAi) is one such mechanisms. It is well preserved in most multicellular eukaryotic groups and maintains sequence-specific regulation of gene expression [1, 2]. Gene silencing is triggered by microRNA (miRNA) or short-interfering RNA (siRNA) produced from double-stranded RNA (dsRNA). These miRNAs and siRNAs play essential activities during the growth and development of plants as well as to develop mechanisms against various biotic and abiotic stresses [1–3]. The production and function of these miRNAs and siRNAs largely depend on three main RNAi protein families known as Dicer-like (DCL), Argonaute (AGO), and RNA-dependent RNA polymerase (RDR) [3, 4]. RNA silencing works within three steps: initiation, maintenance, and signal amplification. The beginning of gene silencing requires the production of dsRNAs that are created from plant-encoded RDRs on a variety of RNA templates [5]. The complementary dsRNA is then processed by the act of RNase III-type enzymes called Dicer (DCR) in animals or Dicer-like (DCL) in plants which alter this dsRNA into siRNA/miRNA, tiny RNAs of 19–31 nucleotides in size [5, 6]. Then, one strand of these miRNAs/siRNAs is attached to AGO protein-containing complexes termed RNA-induced silencing complex (RISC) or multiprotein complex. RISC possesses the endonuclease ability to operate cleavage activity on target mRNAs or DNAs that are complementary/homologous to siRNA/miRNA using AGO's RNaseH-type enzyme property for RNA degradation, translational inhibition, or heterochromatin formation [2, 7, 8]. During the signal amplification stage, RDR enzymes are responsible for the synthesis of dsRNAs from single-stranded RNA (ssRNA) templates to start a new cycle of RNA silencing [4, 9].

The gene members of the DCL family are identified and characterized by the six conserved domains, viz., dead box, helicase, dicer dimerization/Duf283, PAZ (Piwi Argonaut and Zwille), RNase III, and dsRNA binding domain (dsRBD) [6, 10]. AGOs are also known as the special kind of protein family for RNA silencing mechanism located in RISC that cleaves the target mRNAs, and they are characterized by the presence of four functional domains or amino acid motifs, viz., ArgoN/Argo-L, C-terminal PIWI (P-element-induced wimpy testis), PAZ, and MID [11]. The third type of RNA silencing machinery gene is the RDR protein which is highly essential for the RNAi mechanism in fungi, nematodes, and plants [12]. RDR proteins are critically important for the start and increase of the silencing process, and these proteins contain a well-preserved sequence motif like the catalytic *β*′ subunit of DNA-dependent RNA polymerases [13]. This gene family, however, possesses an RNA-dependent RNA polymerase (RdRP) domain and helps to form the dsRNA from single-stranded RNAs (ssRNAs) to start a new cycle of RNA silencing [13, 14].

Different studies have been conducted on different crop plants to investigate the role of RNAi-related gene families. In *Arabidopsis thaliana* (*A. thaliana*), 4 DCL, 10 AGO, and 6 RDR genes were identified [15]. In total, 16 genes in tobacco [16] and 32 genes in rice (*Oryza sativa*) were identified. In rice, the *OsAGO2* gene exhibited particular upregulation in response to salt and drought [15, 17]. 28 genes were identified for each tomato, maize, and coffee [1, 18, 19]. 22 genes were identified in grapevine and pepper [2, 20], 20 in cucumber [21], and 51 in the genome of allopolyploid species of *Brassica napus* [22], and 36 genes both in soybean (*Glycine max*) and in sugarcane [23, 24], 38 genes in foxtail millet [25], 25 in sweet orange [26], and very recently a total of 23 RNAi-related genes have been identified in barley [27].

Successive investigations on RNAi-related regulatory genes in various important crop and fruit plants showed considerable divergence with important roles in different genomic functions. In *A. thaliana* (At), *AtDCL1* mainly plays the role in miRNA biogenesis while *AtDCL2*, *AtDCL3*, and *AtDCL4* mediate siRNA processing [21, 28]. Besides, *AtDCL3* and *AtAGO4* are essential for RNA-directed DNA methylation of the FWA transgene, which is associated with histone H3 lysine 9 (H3K9) methylation [19, 28]. Though *AtDCL2* produces siRNAs to create a defensive mechanism against viral infection and *cis*-acting antisense transcript, *AtDCL4* regulates the vegetative stage transformation by the production of siRNAs from the *trans*-acting transcript [19, 29]. Surprisingly, *AtDCL3* selects short dsRNAs but *AtDCL4* cleaves the long dsRNA substrates [28]. Many other functions of *DCL* genes in plants such as *AtDCL1* and *AtDCL3* genes promote flowering [30]. A recent study has shown that three DCL proteins (Pt-DCL1, Pt-DCL2, and Pt-DCL3) are significantly associated with the *Puccinia triticina* (*P. triticina)* infection which is one of the harmful rust diseases of wheat [31].

On the other hand, AGO genes act as a leading role player in the RNA-mediated gene silencing mechanisms which play a vital role in the growth and development of plants [32, 33]. *AtAGO1* is associated with the transgene-silencing pathways [34] and *AtAGO4* with epigenetic silencing [35]. *AtAGO7* and *AtAGO10* affect growth [36] and meristem maintenance [37]. Other *AtAGO*s however have some important characteristics in gene silencing pathways. Earlier investigations showed that the RDR genes are biologically active in RNAi mechanisms such as cosuppression, protection pathogen invasion, chromatin modification, and post-transcriptional gene silencing activities in plants, viz., *Arabidopsis*, maize [38–40].

However, very limited studies have been conducted regarding RNA silencing machinery genes of wheat crop (*Triticum aestivum*) which is a global common staple food as well as the second most-produced cereal crop after rice in the world. It is also a high vital source of carbohydrates and is the leading source of vegetal protein in human food. So far, only AGO gene families were studied for wheat crops and reported only two genes *TaAGO1b* and *TaAGO4* that might play a substantial role during vegetative and reproductive stages by mediating the cold environment at the vernalization condition [33]. Therefore, a more in-depth study is required on RNAi genes including DCL and RDR gene families for the wheat crop. In this study, an attempt was made to carry out comprehensive genome-wide identification, characterization, and diversity analyses of all members of DCL, AGO, and RDR gene families highlighting their functions, structures, and regulators for the improvement of wheat crops by using the integrated bioinformatics approaches.

## 2. Materials and Methods

### 2.1. Data Sources

Protein sequences of RNAi genes (*DCL*, *AGO*, and *RDR*) for *Arabidopsis* and rice (*Oryza sativa*) were downloaded from TIAR (The Arabidopsis Information Resource) version 10 (http://www.arabidopsis.org) [41] and RGAP (Rice Genome Annotation Project) version 7 (http://rice.plantbiology.msu.edu/) [42] databases, respectively. The *Arabidopsis* RNAi protein (AtDCL, AtAGO, and AtRDR) sequences were used as the query sequences in the basic local alignment search tool (BLAST-P) to explore wheat (*Triticum aestivum*) RNAi genes (*TaDCL*, *TaAGO*, and *TaRDR*) from its genome that was deposited in the PGSB International Wheat Genome Sequencing Consortium (IWGSC) version 1.0 database (http://pgsb.helmholtz-muenchen.de/plant/wheat/iwgsc/index.jsp) plugin in the Phytozome database version 10 (http://phytozome.jgi.doe.gov/pz/portal.html) [43].

### 2.2. Bioinformatic Analysis Approaches

Several bioinformatics analyses were carried out to reach the goal of this study as described in the following subsections.

### 2.3. Identification and Characterization of Wheat RNAi Genes

We considered the protein BLAST (BLAST-P) web tool to identify wheat RNAi genes (*TaDCL*, *TaAGO*, and *TaRDR*) from its genome against the *Arabidopsis* RNAi genes (*AtDCL*, *AtAGO*, and *AtRDR*) as already mentioned in the data sources. The alignment score (≥50), identity (≥50%), coverage (≥50%), *E* values (≥10*E* − 10) [26, 44], and *p* value (<0.001) were utilized to identify the RNAi genes of wheat. We downloaded all the selected RNAi genes from its genome. Then, we performed the multiple sequence alignment (MSA) and maximum-likelihood- (ML-) based phylogenetic tree analyses by using the Clustal Omega (https://www.ebi.ac.uk/Tools/msa/clustalo/) [45] to designate the predicted RNAi gene names (*TaDCL*, *TaAGO*, and *TaRDR*) and their categorization against the RNAi gene families of *Arabidopsis thaliana* and rice. Then, we explored their conserved functional domains by using the web-based Pfam (http://pfam.sanger.ac.uk/), simple modular architecture research tool (SMART, v.8) (http://smart.embl-heidelberg.de/) [46], and the National Center for Biotechnology Information Conserved Domain Database (NCBI-CDD; http://www.ncbi.nlm.nih.gov/Structure/cdd/wrpsb.cgi). Some basic information about these genes such as the accession number, genomic location, gene length, and encoded protein length was downloaded from the Phytozome database. The molecular weight of each gene member of the DCL, AGO, and RDR groups was predicted by using the online tool ExPASyComputepI/Mwtool (http://au.expasy.org/tools/pitool.html).

### 2.4. Conserved Functional Domain and Motif Analysis

The conserved functional domain analysis of predicted RNAi genes (*TaDCL*, *TaAGO*, and *TaRDR*) was carried out based on Pfam, SMART, and NCBI-CDD databases. Also, to identify the most potentially conserved metal-chelating catalytic triad regions or residues in the PIWI and RdRp domains, proteins of TaAGO and TaRDR families were considered. The alignment profiles were produced by the Clustal Omega and GENEDOC v2.6.002 software [47]. All paralogs of AtDCL, AtAGO, and AtRDR protein families were considered during the alignment. Moreover, the conserved structural motif differences among TaDCL, TaAGO, and TaRDR protein groups were predicted using a bioinformatics tool known as multiple expectation maximization for motif elicitation (MEME-Suite v5.3.3) [48]. The MEME analysis was carried out by specifying the parameters as (i) optimum motif width as ≥6 and ≤50 and (ii) maximum 20 motifs.

### 2.5. RNAi Gene Structure and Chromosomal Distribution

The predicted RNAi gene (exon-intron) structures were analyzed using the gene structure display server (GSDS) (2.0: http://gsds.cbi.pku.edu.cn/index.php) [49] against the coding sequences (CDS) and DNA sequences of 4 *AtDCL*, 10 *AtAGO*, and 6 *AtRDR* genes. Additionally, the genomic locations and duplication events of the DCL, AGO, and RDR gene families in wheat were investigated using the web-based tool MapGene2Chromosome V2 (http://mg2c.iask.in/mg2c_v2.0/) [50].

### 2.6. RNAi Protein Structure and Nonsynonymous/Synonymous Mutation Ratio

The three-dimensional (3D) protein structure analysis was also carried out by using the SWISS-MODEL for all predicted RNAi proteins. Also, the calculations of synonymous mutations/substitutions (Ks), nonsynonymous mutation/substitution (Ks) rates, and nonsynonymous/synonymous mutation ratio (Ka/Ks) were performed for all genes using Clustal Omega (https://www.ebi.ac.uk/Tools/msa/clustalo/) [45] and PAL2NAL bio tool v14.0 (http://www.bork.embl.de/pal2nal/) [51]. If the ratio Ka/Ks = 1, then, it was called neutral selection, it was called positive selection (adaptive evolution) if Ka/Ks > 1, and it was called purifying/negative (conserved) selection if Ka/Ks < 1.

### 2.7. Subcellular Localization Analysis

A web-based tool called PSI (plant subcellular localization integrative predictor) (http://bis.zju.edu.cn/psi/) and open-source software R-3.5.2 were utilized for predicting the subcellular location of the predicted RNAi genes (*p* value < 0.05).

### 2.8. *Trans*-Regulatory Elements (TREs) of the Predicted RNAi Genes

To identify key transcription factors (TFs) as the transcriptional regulatory factors (trans-acting) of the predicted RNAi genes, we performed RNAi genes versus TF interaction network analysis based on the plant transcription factor database (plantTFDB v4.0) (http://planttfdb.gao-lab.org/) [52]. We constructed both RNAi gene regulatory networks and subnetworks to find out the leading TFs and hub-RNAi genes using Cytoscape 3.7.1 [53].

### 2.9. *cis*-Regulatory Elements (CREs) of the Predicted RNAi Genes

To find the *cis*-acting regulatory elements (CAREs) in the promoter, enhancer, and silencer regions of the predicted RNAi genes, we considered a web-based bioinformatics tool PlantCARE (http://bioinformatics.psb.ugent.be/webtools/plantcare/html/) [54].

### 2.10. Expression Analysis of the Predicted RNAi Genes

To investigate the expressions of the predicted RNAi genes at various organs or tissues in different conditions/environments, expressed sequence tag (EST) analysis was carried out from PlantGDB (http://www.plantgdb.org) [55] at various tissues and organs of wheat. The heat map was created using R-3.5.2. Additionally, the expression analysis was performed against drought and heat stress by using the WheatExp database [56]. The CDS for each of the predicted RNAi genes was used to extract expression data, and a web-based Heatmapper [57] tool was used to visualize the heat map.

## 3. Results and Discussion

### 3.1. Identification and Characterization of RNAi Genes

**Figure 1:**
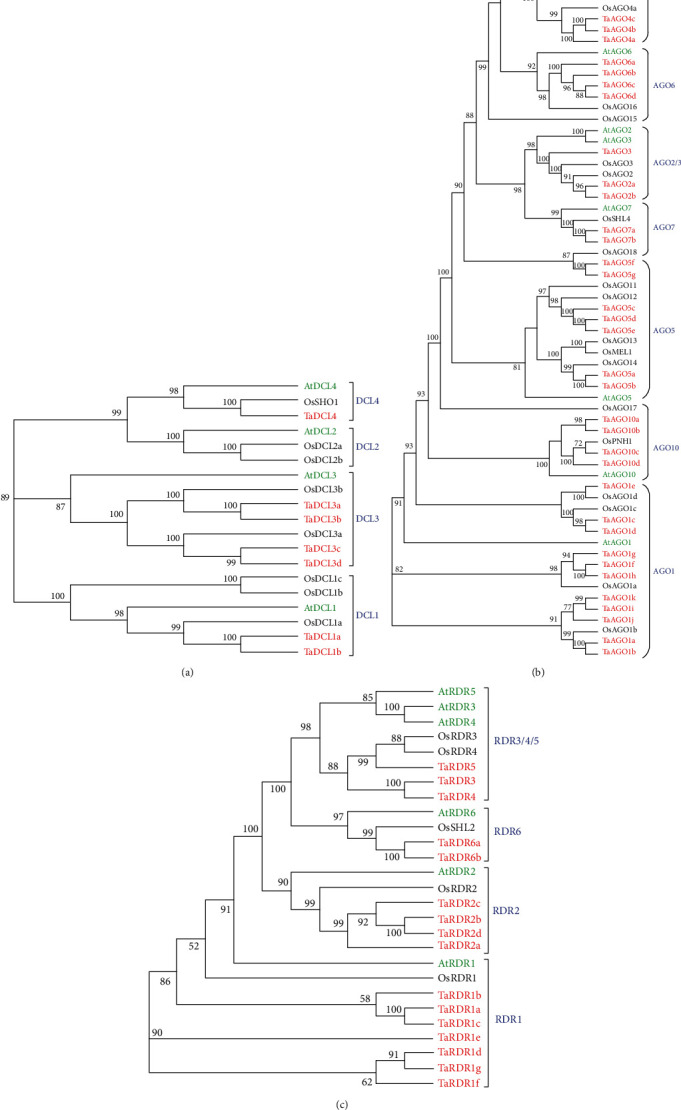
Phylogenetic trees based on the RNAi-related proteins of wheat, rice, and *Arabidopsis.* Maximum-likelihood- (ML-) based phylogenetic trees (a) DCL, (b) AGO, and (c) RDR were produced by using the Clustal Omega.

To select the best possible RNA silencing genes (*TaDCL*, *TaAGO*, and *TaRDR*) from the wheat genome, the *Arabidopsis* RNA silencing protein sequences of 4 AtDCLs, 10 AtAGOs, and 6 AtRDRs were used as the query sequences for BLAST-P search against the wheat genome in the Phytozome database as described in Materials and Methods. Then, we validated the selected TaDCL proteins based on six well-known conserved domains (DExD-helicase, helicase-C, Dicer-dimer/Duf283, PAZ, and RNase III (Ribonuclease-3) and double-stranded RNA binding (dsRB/DSRM)) of AtDCL proteins [15, 19, 58], TaAGO proteins based on two well-known conserved domains (PAZ and PIWI) of AtAGO proteins [2], and TaRDR proteins based on the widely used RdRp domain of AtRDR proteins [2] by using three databases Pfam, NCBI-CDD, and SMART (Table S1). Thus, in total, 62 RNAi-related genes (7 *TaDCL*, 39 *TaAGO*, and 16 *TaRDR*) were identified for subsequent analyses. To understand the evolutionary relationship of these three predicted RNAi gene groups corresponding to the *Arabidopsis* and rice homologs, three independent phylogenetic trees (Figure 1) were constructed based on their protein sequences (Data S1-S3). Figure 1(a) showed that the 7 TaDCLs are classified into four distinct groups. Based on phylogenetic relatedness and sequence homology with 4 AtDCLs, the TaDCLs were named TaDCL1a, TaDCL1b, TaDCL3a, TaDCL3b, TaDCL3c, TaDCL3d, and TaDCL4. There was no TaDCL2 protein(s) corresponding to AtDCL2 and OsDCL2. Similarly, the 39 TaAGOs were consisted of 11 TaAGO1s, 2 TaAGO2s, one TaAGO3, 3 TaAGO4s, 7 TaAGO5s, 5 TaAGO6s, 2 TaAGO7s, one TaAGO8, 3 TaAGO9s, and 4 TaAGO10s of 7 clusters (Figure 1(b)). It was observed from the tree that TaAGO8 and 3 TaAGO9s formed a group with the nearest taxa OsAGO4b, not with the corresponding AtAGO8 and AtAGO9. On the other hand, it was obvious that TaAGO5a and TaAGO5b were grouped with OsAGO14. The three proteins TaAGO5c, TaAGO5d, and TaAGO5e formed a group with OsAGO11 and OsAGO12. The two proteins TaAGO5f and TaAGO5g specifically clustered with taxa OsAGO18 but not with any AtAGOs. The clustering trend of these 11 TaAGOs with several rice RNAi-related AGO proteins indicated that they contained monocot-specific genetic characteristics that had originated during the evolutionary process [15, 19]. Like TaDCLs and TaAGOs, the TaRDR proteins were named based on the AtRDRs homologs. Figure 1(c) showed that the phylogenetic tree of 16 TaRDR proteins with 6 AtRDR and 4 OsRDR proteins produces four major clusters. We observed that the TaRDR1 clade contained 7 members corresponding to the AtRDR1 and OsRDR1 and the TaRDR2 clade contains four members corresponding to the AtRDR2 and OsRDR2 homologs. The three proteins TaRDR3, TaRDR4, and TaRDR5 formed a cluster corresponding to the AtRDR3, AtRDR4, AtRDR5, OsRDR3, and OsRDR4 proteins. The clade TaRDR6 consisted of TaRDR6a and TaRDR6b, which produces a distinct group with AtRDR6 and OsSHL2 proteins.

According to the Phytozome database, the largest gene sequence length of the predicted *TaDCL* members was found at 14743 bp for *TaDCL4* and the smallest length of 8347 bp for *TaDCL3d* with their corresponding protein sequence lengths being 1392 and 1586 amino acids (Table 1). *TaDCL1a*, *TaDCL1b*, and *TaDCL4* might be indispensable for the biogenesis of 21 nt small RNAs that correspond to miRNAs and tasiRNAs since they are homologs with *AtDCL1* and *AtDCL4* [15]. *TaDCL3a*, *TaDCL3b*, *TaDCL3c*, and *TaDCL3d* genes might play a vital role to produce the 24 nt RNAs that mediate *de novo* DNA methylation and gene silencing and chromatin modification like their homolog *AtDCL3* gene [58]. Our predicted *TaDCL* genes may also act to direct cleavage of positive-sense RNA viruses such as *Cucumber mosaic virus* (a cucumovirus), *Oilseed rape mosaic virus* (a tobamovirus), and *Turnip crinkle virus* (a carmovirus) like their homolog gene *AtDCL3* [29, 59]. Hence, the homolog of the *AtDCL3* gene group in wheat such as *TaDCL3a*, *TaDCL3b*, *TaDCL3c*, and *TaDCL3d* may provide a significant role in gene silencing against viral infection in wheat like *Arabidopsis*.

Argonaute proteins are known as very important RNA binding proteins. Based on the two domains (PAZ and PIWI) characteristics [2], a total of 39 TaAGOs were identified from the wheat genome (Table 1). Domain analysis using Pfam, SMART, and NCBI-CDD showed that all TaAGO proteins contain an N-terminus PAZ domain and a C-terminus PIWI domain. TaAGOs possessed some other functional domains like *A. thaliana* RNAi-related proteins, viz., ArgoN, ArgoL1, DUF1785, ArgoL2, and ArgoMid (Table S1). The largest gene sequence length of the *TaAGO* genome was found to be 12443 bp for *TaAGO1g* and the smallest gene length was 3234 bp for *TaAGO3* with their corresponding protein lengths of 1085 and 849 amino acids (Table 1). The protein members of the TaAGO family are predicted to contribute significantly to RNA-directed gene suppressing actions and are vitally engaged in the developmental processes at various organs or tissues in wheat like other plants [32, 60, 61]. Also, some earlier investigations suggested that the PIWI domain in AGO proteins presents complete homology to RNase H that binds the siRNA 5′ end to the target RNA [62] and takes part to cut target mRNAs that display sequences complementary to siRNA or miRNAs [63]. Argonautes play as catalytic proteins that are known to contain three conserved metal-chelating residues/regions in the PIWI domain such as aspartate (D), aspartate (D), and histidine (H) called DDH [15] that act as the catalytic triad. This triad was originally explored in AtAGO1.

Finally, a total of 16 TaRDR proteins were found to possess an RdRp domain (Table S1). The gene length of the *TaRDR* genes varied from 15893 bp for *TaRDR2b* to 1707 bp for *TaRDR1d* with their corresponding encoding potential of 1127 aa and 484 aa (Table 1).

### 3.2. Analysis of Conserved Domains and Motifs for Predicted RNAi Proteins

**Figure 2:**
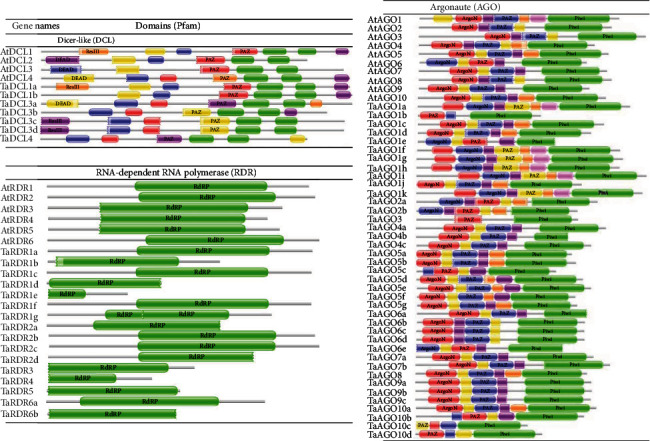
Domain composition of TaDCL, TaAGO, and TaRDR protein families. Conserved functional domains were predicted from the Pfam database. Domains are indicated in different colour boxes with their corresponding names inside the box.

Protein-conserved domains play a vital role in protein-protein interactions (PPI), enzymatic activity, DNA binding, and other crucial cellular processes. In this study, members of the TaDCL, TaAGO, and TaRDR protein groups were selected from three databases based on the highest number of conserved domains. According to the referenced conserved domains as mentioned previously, six functional domains, namely, DEAD/ResIII, Helicase-C, Dicer-Dimer, PAZ, RNase III, and DSRM, were taken into consideration to select the final set of putative *TaDCL* genes. It was observed from the domain search results with three databases that TaDCL proteins possessed almost all of these six common referenced domains (Table S1 and Figure 2). These domains are known as the highly functional plant DCL domains for the protein structure [15, 19, 28, 58]. Ribonuclease-3 (RNase III) domain characteristics of TaDCL proteins enable them to cleave dsRNA to generate small interfering RNAs (siRNAs) that play crucial functions to regulate gene expression in plants [19, 28, 64]. The TaRDR proteins were selected with the presence of RdRP and RRM functional domains that were also considered by [2] [2] in identifying the RDR gene. We observed that the wheat genome possesses 16 RDR proteins that share a mutual motif analogous to the catalytic *β*′ subunit of RNA-dependent RNA polymerases (RdRp) (Table S1 and Figure 2), which was also supported by a previous study [65]. The RdRp is one of the key multipurpose enzymes of RNA viruses essential to replicate the genome and execute transcription. The RDR1 and RDR2 proteins present a substantial impact on siRNA biogenesis and promote the RNAi mechanism [26].

The TaAGO proteins that contained Argo-N/Argo-L, PAZ, MID, and PIWI functional domains were termed as AGO class proteins in wheat according to the suggestion of [2] [2]. The Argonaute proteins usually possess a Piwi-Argonaute-Zwille (PAZ) domain and a PIWI (P-element induced wimpy testis) domain [66]. The domain analysis results in Table S1 and Figure 2 showed that all 39 predicted TaAGOs also hold these two most common domains. The N-terminus PAZ, which is responsible for small RNA binding, and the C-terminus PIWI domain perform catalytic activities similar to that of *Arabidopsis* and rice [15, 67]. Moreover, a domain (DUF1785) was observed before the PAZ domain in all TaAGO proteins except in TaAGO1b, TaAGO10c, and TaAGO10d. The nonexistence of this domain might occur due to the loss of its N-terminal sequence during evolution. AGO protein members are associated with siRNA and miRNA maturation. They maintain chromosomal integrity and contribute to the generation of a new group of small noncoding RNAs called Piwi-interacting (pi) RNAs [67]. The PAZ and PIWI domains in AGOs are the main functional domain that produces RISC for gene silencing in all species [15, 68]. The PIWI domain has a catalytic triad of three residues aspartate (D), aspartate (D), and histidine (H) which is known as DDH [15]. Some previous studies reported that the increasing number of aspartate (D) family is extremely essential to maintain the nutritional properties in maize grains like other plants, especially in seeds, because of its vital role in the synthesis of four important amino acids such as lysine/Lys (K), threonine/Thr (T), methionine/Met (M), and isoleucine/Ile (I) [69–71]. Histidine/His (H) is one of the important amino acids that play a substantial role during plant growth and development [72]. The physiological investigation also showed that histidine plays novel functional activities in plants as chelators and transporters of metal ions [73].

**Figure 3:**
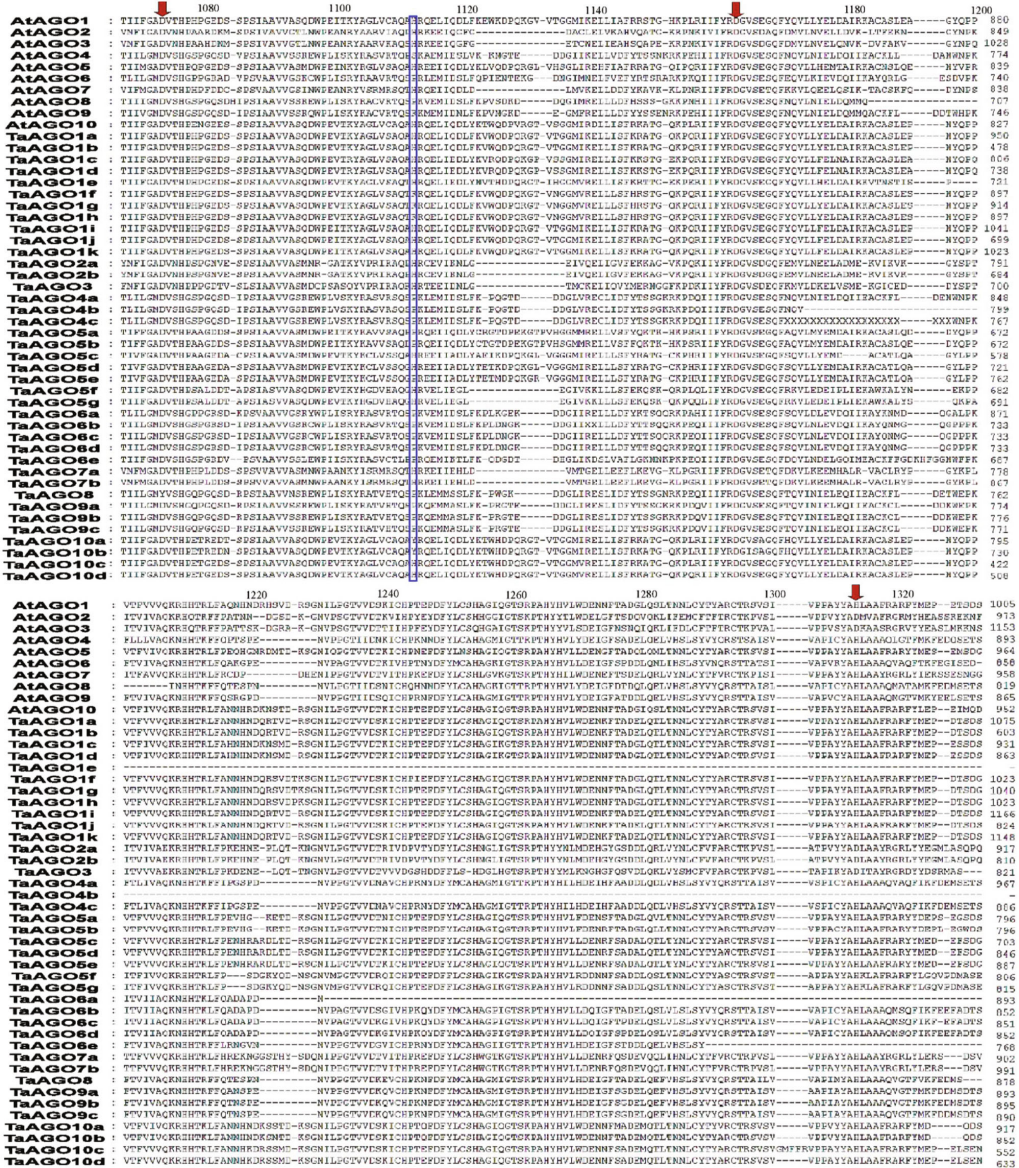
Alignment profile of PIWI domain amino acids of TaAGO proteins. The protein sequences were aligned using Clustal Omega. The conserved Asp, Asp, and His (DDH) triad residues corresponding to D760, D845, and H986 of AtAGO1 are indicated with downward arrows, whereas the conserved H residue corresponding to H798 of AtAGO1 is boxed with a blue colour. Amino acid positions corresponding to each protein are indicated at the end of each line.

The multiple sequence alignment (MSA) showed that 15 TaAGO proteins (out of 39) including 7 TaAGO1s (TaAGO1a-d, TaAGO1i-k), 4 TaAGO5s (TaAGO5c-d, TaAGO5f-g), 2 TaAGO7s (TaAGO7a-b), and 2 TaAGO10s (TaAGO10c-d) possessed the triad residues (DDH/H) similar to those of *Arabidopsis*. The rest of the 24 TaAGO proteins showed at least one variation among these catalytic triad residues (Figure 3 and Table 2). The triad residue aspartate (D) at the 760^th^ position (D760) in TaAGO8 was altered by tyrosine (Y). Furthermore, tyrosine (Y) was conserved instead of histidine at position H798 in TaAGO10a and TaAGO10b proteins. The tyrosine/Tyr (Y) residue plays a key role in protein phosphorylation which is an essential regulatory mechanism that maintains numerous biological processes and molecular functions in plants [74].

The triad residue histidine (H) at position H986 was missed (-) in TaAGO1e, TaAGO4b, TaAGO6a, and TaAGO6e, as well as the fourth residue histidine (H) at position H798 in TaAGO4b, TaAGO6a, and TaAGO6e, was replaced by the proline (P). The triad residue histidine (H) at position (H798) of 2 TaAGO4s (TaAGO4a and TaAGO4c), 3 TaAGO5s (TaAGO5a, TaAGO5b, and TaAGO5e), 3 TaAGO6s (TaAGO6b, TaAGO6c, and TaAGO6d), and 3 TaAGO9s (TaAGO9a, TaAGO9b, and TaAGO9c) was also replaced by the proline (P). The proline (P) residue in AGO protein plays a vital role in plants against different stress conditions including metal chelator, antioxidative defense, and signalling molecules [75, 76]. The triad residue H at position H798 of TaAGO1f, TaAGO1g, and TaAGO1h was replaced by arginine (R). The arginine/arg (R) residue plays a vital role in plants for different physiological and biochemical processes, growth and development, and adaptation against various environmental factors [77]. Thus, the replacement/mutational changes of the D760, D845, H986, and H798 (DDH/H) catalytic residues in the TaAGO proteins may also play vital roles to improve the wheat plants' growth and development as well as the adaptation ability against different biotic and abiotic stress.

Motifs in DNA/RNA/protein sequence encode different biological functions [48]. Their identification and characterization play an important role to understand the gene/protein functions. These motifs are also known as the small active site of an enzyme for accurate folding of the protein and molecular evolution [48]. In this work, multiple EM for motif elicitation (MEME) was used to predict motifs for each member of the TaDCL, TaAGO, and TaRDR protein families against 20 motifs of *Arabidopsis*. It was observed that most of the predicted motifs were conserved in each of the TaDCL proteins according to the similar order of AtDCLs (Figure S2). The TaDCL4 possessed some C-terminal motifs similar to that of the AtDCL4 which was also supported by an earlier investigation [19]. All TaDCL proteins possessed 20 conserved motifs except TaDCL3b and TaDCL4 which contained 19 and 16 motifs, respectively (Figure S2).

On the other hand, out of 39 TaAGO proteins, a total of 28 proteins were composed of 16–20 conserved motifs and the remaining 11 proteins were composed of 10–15 motifs. High conservation was found in TaAGO1, TaAGO9, TaAGO10, TaAGO1b, TaAGO1e, and TaAGO10c proteins with motif sizes 14–20 (Figure S2). Motif search for the TaRDR proteins revealed that the predicted 10 TaRDRs possessed 17–20 highly conserved functional motifs in their RdRp domains similar to that of the AtRDR protein members (Figure S2), but TaRDR1e, TaRDR3, TaRDR4, and TaRDR5 possessed only 7–8 conserved motifs. Thus, the motif analysis also suggested that there is a tendency for high conservation of functional motifs in the respective domains similar to that of the AtDCL, AtAGO, and AtRDR proteins. Hence, the predicted motifs might be useful to understand different biological functions including the cellular processes at the molecular scale to identify the mechanisms of diseases in wheat similar to that of the other eukaryotic groups [48].

### 3.3. RNAi Gene Structure Analysis

**Figure 4:**
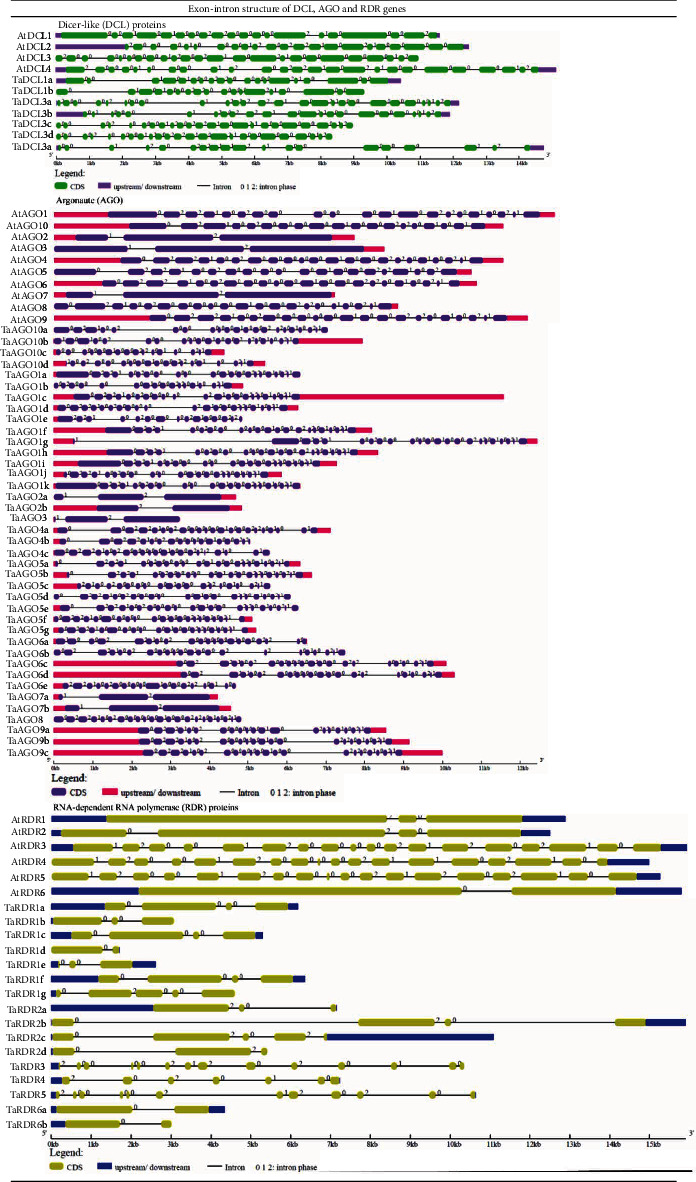
Gene structure of wheat RNAi-related genes corresponding to *A. thaliana* DCL, AGO, and RDR genes. The exons (green/violet/light green colour box), introns (black lines), and intron phases (0, 1, and 2) were mentioned.

The exon-intron organization of the predicted RNAi genes was investigated by using the GSDS2.0 web tool for understanding their probable structural patterns and the level of similarity with the respective *Arabidopsis* RNAi genes. Our findings showed that intron and exon distribution and their proportion are almost similar to those of the respective *Arabidopsis* RNAi genes (Table 1 and Figure 4). The intron numbers of *TaDCL*s varied from 17 to 25 while for *AtDCL*s, the range was 11–17. On the other hand, *TaAGO* genes possessed almost the same number of introns as their *AtAGO* counterpart. The intron numbers varied from 14 to 22 and 18 to 21 in wheat and *Arabidopsis* AGO genes, respectively.

Among the TaRDR genes, *TaRDR3* contained the highest 13 introns followed by 11 in *TaRDR5*. The range for the other genes was 1–6. The same trend was also observed in *Arabidopsis*. An earlier investigation showed that RDR genes play significant roles in plants for gene silencing against viral diseases, for example, *CaRDR1* possessed an important role in pepper resistance against TMV [78]. It was observed that the exon-intron structures within the subgroups of *TaDCL*, *TaAGO*, and *TaRDR* genes groups were very similar. Thus, we observed that the predicted RNAi gene structures are almost similar to the structures of the respective *Arabidopsis* RNAi genes.

### 3.4. Chromosomal Mapping for the Predicted RNAi Genes

**Figure 5:**
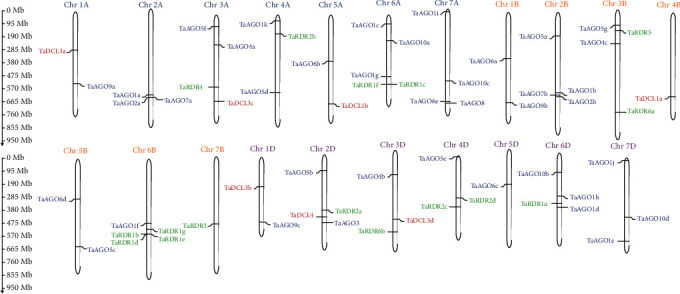
Visualization of *TaDCL*, *TaAGO*, and *TaRDR* gene localization on all 21 chromosomes. Chromosome numbers are displayed at the top of each vertical bar. The gene names on the left and right sides of each chromosome correspond to the approximate locations of each RNAi-related gene. The scale on the left is in megabases (Mb).

The genomic distributions of the 7 *TaDCL*, 39 *TaAGO*, and 16 *TaRDR* genes at 21 different chromosomes in the three genome groups A, B, and D of wheat were visualized in Figure 5 by using the MapGene2Chrom v2 web tool. The predicted map showed that all identified 62 RNAi genes in wheat were spread across all chromosomes by containing 1–5 RNAi genes in each chromosome. Exactly one gene in chromosome 4B (*TaDCL1a*), 7B (*TaRDR3*), and 5D (TaAGO6c) and five genes in Chr6A (*TaAGO1c*, *TaAGO1g*, *TaAGO10a*, *TaRDR1f*, and *TaRDR1c*) and Chr6B (*TaAGO1f*, *TaRDR1b*, *TaRDR1d*, *TaRDR1e*, and *TaRDR1g*) were found. Seven *TaDCL*s, however, were segmentally distributed in seven distinct chromosomes of which 3 genes (*TaDCL3a*, *TaDCL3c*, and *TaDCL1b*) were in genome group A (Chr1A, Chr3A, and Chr5A) and 1 gene (*TaDCL1a*) in genome group B (Chr4B), and the rest of the 3 genes (*TaDCL3b*, *TaDCL4*, and *TaDCL3d*) were located in genome group D (Chr1D, Chr2D, and Chr3D). Moreover, each of the 19 chromosomes contained at least 1 *TaAGO* gene but the rest of the 2 chromosomes (Chr4B, Chr7B) did not contain any *TaAGO* genes. Out of 39 *TaAGO* genes, 16 genes were found in 7 chromosomes of genome group A, 11 genes in 5 chromosomes of the B genome group, and 12 genes in 7 chromosomes of the D genome group. Genomic distribution of the 16 *TaRDR* genes was found within 10 chromosomes of which 4 TaRDR genes *TaRDR4*, *TaRDR2b*, and *TaRDR1f*-*c* (with tandem duplication) were located in chromosomes Chr3A, Chr4A, and Chr6A, respectively, of the A genome group. Out of another 5 *TaRDR* genes, *TaRDR3* was found in Chr7B and two pairs of genes *TaRDR1b*-*d* and *TaRDR1e*-*g* (with tandem duplication) belonged to chromosome Chr6B.

It should be noted here that tandem gene duplication is defined as the successively occurring homologous genes (BLAST *E* value < 1*E* − 20) and not being disrupted by nonhomologs [79]. Based on these characteristics, we found that *TaAGO1h* and *TaAGO1d* also experienced tandem duplication events. These results suggested that gene duplication events might contribute to the expansion of the *TaRDR* and *TaAGO* gene families. Evolutionarily the RNAi-dependent pathway genes showed some segmental and tandem duplication events for the expansion of these genes in the *Brassica napus*, *Zea mays*, and *Sorghum bicolor Oryza sativa* [15, 19, 22].

### 3.5. Structural Analysis of Proteins and Ka/Ks Ratio Calculations

The two most common secondary/3D structural elements of the protein sequence are the alpha helix (*α*-helix) and the beta-sheet (*β*-sheet)/beta-strand which are associated with different biological functions [80]. The structural analysis of the predicted TaDCL proteins exhibited almost similar structures though some differentiations were observed in TaAGO and TaRDR protein sets (Figure S3A and Table S2). It was predicted that they were conserved and engaged to perform similar functions. Obtained results suggested that the RNAi-related proteins in wheat possessed *α*-helix and *β*-folding, belonging to a hybrid protein structure and forming the appropriate place to perform actions for synthesizing the particular binding pocket. This pocket anchors the characteristics of a two-nucleotide 3′ overhang that results from the digestion of RNAs by RNase III for producing miRNA/siRNAs [81]. The average alpha-helix and *β*-folding for TaDCL proteins were 11 and 18, respectively. The highest alpha-helices 14 and the highest *β-*sheets 26 were related to TaDCL1a and TaDCL3d (Table S2).

The AGO proteins in wheat typically possessed 18 and 14 alpha-helices and *β-*sheets. The maximum alpha-helices 26 found in TaAGO5b and the maximum *β-*sheets 16 were found in six TaAGO proteins (TaAGO1a, TaAGO1c, TaAGO1j, TaAGO5c, TaAGO5f, and TaAGOg) (Table S2). An earlier investigation also showed that the AGO proteins of 32 plants possessed different numbers of alpha-helix and *β-*sheet that implied a hybrid protein structure [81]. On the other hand, the RDR protein sequences possessed on average 12 and 14 alpha-helices and *β-*sheets with the highest *α*-helix by TaRDR1b and the highest *β*-folding by TaRDR1a. Interestingly, the protein TaRDR1e contained one alpha-helix and 3 *β-*sheets followed by TaRDR4 that displayed 6 and 8 alpha-helices and *β-*sheets and it might have occurred for the small size of two proteins of 333 and 543 aa (Table 1 and Table S2).

The synonymous sites (S), nonsynonymous sites (K), synonymous substitution/mutation rates (Ks), nonsynonymous substitution/mutation rates (Ka) of DNA base pairs, and the ratio Ka/Ks were estimated by the online tools Clustal Omega and PAL2NAL. PAL2NAL calculates Ks and Ka by codeml in the phylogenetic analysis by maximum likelihood (PAML) package [82]. The ratio (Ka/Ks) calculation results were <1 (Table S2) for all genes of *TaDCL*, *TaAGO*, and *TaRDR* groups implying that there were more synonymous changes than nonsynonymous changes. This suggested that the structural forms of the predicted RNAi proteins in groups TaDCL, TaAGO, and TaRDR are almost similar to the corresponding RNAi proteins in groups AtDCL, AtAGO, and AtRDR, respectively.

### 3.6. Subcellular Location of the Predicted RNA-Related Genes

**Figure 6:**
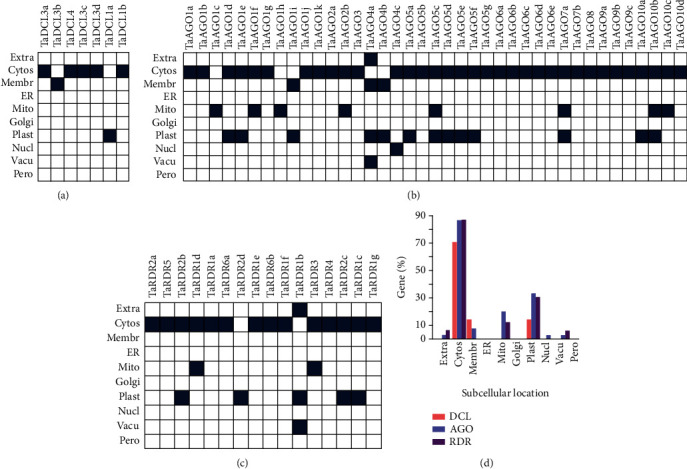
Prediction of subcellular locations of *TaDCL*, *TaAGO*, and *TaRDR* gene families. The prediction was made for cytosol (cytos), endoplasmic reticulum (ER), extracellular (extra), Golgi apparatus (Golgi), membrane (membr), mitochondria (mito), nuclear (nucl), peroxisome (pero), plastid (plast), and vacuole (vacu) for each of the (a) DCL, (b) AGO, and (c) RDR gene families with the help of PSI and R-3.6.3.

Subcellular location (SCL) analysis revealed that most of the RNA silencing genes were located in the cytosol (DCL is 71.4%, AGO is 87.2%, and RDR is 87.5%) followed by plastid (DCL is 14.3%, AGO is 33.3%, and RDR is 31.2%) (Figure 6 and Table S3). Degradation of specific mRNAs to reduce the specific gene expression in plants commonly occur in the cytoplasmic organs, which implied that the predicted RNAi genes/proteins are closely involved in the PTGS activities [83]. In PTGS, the RNAi proteins largely participate in the RNA-induced silencing complex- (RISC-) mediated cleavage activities with the substrate molecules [84]. The cytosol is the place where maximum metabolism in plants happens and most of the proteins in the cell are located in the cytosol [84]. It may assume that the identified proteins in wheat are placed in cytoplasmic organelles that are responsible for essential chemical activities and energy transformations connected to wheat plant growth, repair, and reproduction. Some TaAGOs (20.5%) and TaRDRs (12.5%) were found in mitochondria. The genes found in mitochondria might play the role of integrators of signals and take part in both development and stress response pathways [85].

### 3.7. *Trans*-Regulatory Elements (TREs) of the Predicted RNAi Genes

A transcription factor (TF) also known as a *trans*-acting factor interacts with a particular *cis*-acting component in the promoter site to regulate RNAi genes in plants and other eukaryotic groups [86]. In this study, 375 TFs were identified that may regulate the identified RNAi genes in wheat (Data S5 and Figure S4). The identified TFs were classified into 27 groups/families. The top-ranked 9 TF families (ERF, MIKC-MADS, C2H2, BBR-BPC, MYB, Dof, LBD, CPP, and AP2) consisted of 157 (41%), 38 (10%), 34 (9%), 18 (5.8%), 17 (4.5%), 15 (4%), 15 (4), 10 (2.6%), and 10 (2.6%) regulators, respectively, that is 314 (83%) in total out of 375 (Table S4 and Figure S5).

The TF family ERF (ethylene response factor) is one of the largest subfamilies that belong to the APETALA2/ERF family. It includes ethylene signalling and the response pathway in plants that was characterized by a single AP2 domain [87]. This TF family also responds to plant hormones with improved plant survival during stress conditions. For example, several AP2/ERF families respond to the plant hormones abscisic acid (ABA) and ethylene (ET) to help stimulate ABA and ET-dependent and independent stress-responsive genes [88]. An experimental investigation showed that an ethylene response factor (SlERF5/ERF5) helps to increase adaptation to drought and salt tolerance in tomato [89].

The MIKC-MADS family encodes the TFs for important and numerous functions connected to plant growth and development [90]. This TF is popular to act as a regulatory network for rapid and simultaneous functional divergence in vegetative and reproductive stages in plants for regulating gene expression in flowers, pollen, endosperm, guard cells, roots, and trichomes [90]. This family was also responsible for the transcription of *OsRDR1* genes to increase the resistance power against the rice stripe virus (RSV) in rice [26, 91]. C2H2, a zinc finger-type protein, is also one of the influential TF families that possess finger-like structures and can bind Zn^2+^ [92]. This TF plays a vital role in plant growth, development, and stress signal transduction [92, 93]. A study showed that the genes of this TF family play a deep role in salt, osmotic, drought, cold, drought, oxidative, and high-light stress [92]. Some stress-associated plant hormones such as abscisic acid (ABA), salicylic acid (SA), jasmonic acid (JA), and ethylene (ET) perform a crucial role against many environmental stressors (pathogens and abiotic) mediated by C2H2 class proteins [92].

The plant-specific Barley B recombinant-basic PentaCysteine (BBR/BPC) TF family shows essential functions for proper plant growth and development [94, 95]. The proteins of this family regulate flower development, the size of the stem cell niche, and seed development through transcriptional regulation of homeotic transcription factor genes [95]. BBR/BRP also works for brassinosteroid hormone signalling in plants such as *Arabidopsis*. BPC6 targets the promoters of all key brassinosteroid signalling elements [95].

In plants, MYB TF was also found in higher numbers. In *A. thaliana*, this TF family accounts for nearly 9% of the total TFs [96]. This TF was identified for its conserved MYB domain (a 52 amino acid motif) at the N-terminus and was an evolutionarily conserved domain found in almost all eukaryotes [96, 97]. The TFs of this family in plants are also linked to various biological processes, namely, the circadian rhythm, defense and stress responses, cell fate and identity, seed and floral development, and regulation of primary and secondary metabolism [96, 98].

The DNA-binding one finger (Dof) is also a plant-specific TF gene family found in green algae to higher plants which showed bifunctional binding characteristics with DNA and proteins to control transcriptional machinery in plant cells [99, 100]. Dof is involved in regulating genes related to seed maturation and germination, phytohormone and light-mediated regulation, and plant tolerance to biotic and abiotic stresses [99–101]. The proteins of the LBD (lateral organ boundaries domain) TF family found in various plants suggested that the genes play important roles in the regulation of growth and development [102]. Of the two classes of LBD proteins, proteins of class I mostly engaged in organ separation, lateral organ development [102, 103], and auxin signal transduction lead to the development of lateral roots [104] whereas LBD genes in class II are involved in metabolism, specifically as suppressors of anthocyanin synthesis in plants [105]. Several investigations explored some extra activities of the proteins of the LBD TF family in pollen development and plant regeneration, photomorphogenesis, pathogen resistance, and nitrogen metabolism [106]. LBD genes found in *Arabidopsis thaliana* also showed that they had defensive responses against multiple pathogens [102, 106]. Though largely present in different species, to date, a total of 112 CPP (cysteine-rich Polycomb-like protein) families from 16 plants were identified of which TSO1 and CPP1 were identified in *A. thaliana* and soybeans [107]. Some studies showed that TSO1 was largely expressed in flowers and CPP1 was linked to the control of expression of soybean leghemoglobin gene Gmlbc3 [108, 109]. Other than these influential TF families, AP2 was also related to 10 genes and bZIP was related to *TaAGO2b* to respond to important physiological and biochemical stimuli in wheat for regulating gene expression (Figure S5). The earlier investigation also reported that both AP2 and bZIP TF families contributed to gene regulation for development and resistance response to various environmental stressors [86, 110].

**Figure 7:**
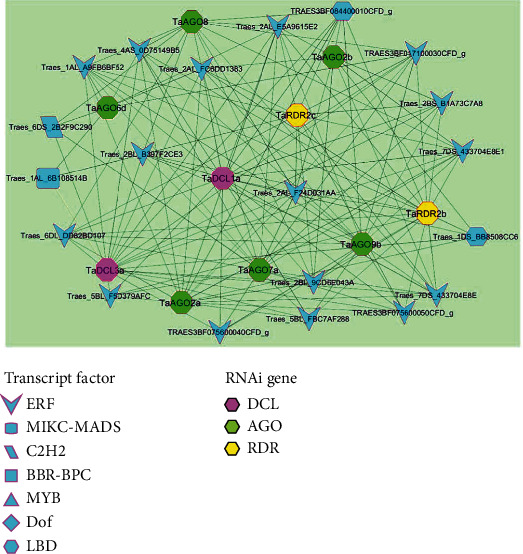
RNAi gene-mediated subnetwork among the hub TF families that regulate three or more RNAi-related genes in wheat.

Additionally, four hub TF families were selected based on the node degree criterion that had three or more connections with the predicted genes. Among them, 16 TFs belong to the ERF family, one TF from MIKC-MADS and C2H2, and two TFs from the LBD family (Figure 7). The ERF family with accession no. Traes_2AL_E5A9615E2 regulates all 10, and a TF of the ERF family with accession number Traes_5BL_F5D379AFC regulates a minimum 5 hub genes in the network followed by the two TF of family LBD with accession no. TRAES3BF084400010CFD_g and Traes_1DS_BB8508CC6 that regulate 8 and 5 genes, respectively (Figure 7). The TF family C2H2 and MIKC-MADS each regulate 4 and 3 genes in the network (Figure 7). It was observed in Figure S6(A-C) that the ERF, MIKC-MADS, and C2H2 TF families largely bind to the 17–19 hub RNAi-related genes. It implied that TFs from these three families showed a high tendency to bind with genes for particular gene expression in wheat. The TF families BBR-BPC, MYB, and Dof bind 7–9 RNAi hub genes (Figure S6(D-F)).

In Figure 7, it was clear that all hub TF families were associated with 10 genes, viz., two genes from the *TaDCL* family (*TaDCL3a* and *TaDCL1a*), 6 genes from the *TaAGO* family (*TaAGO2a*/*2b*, *TaAGO6d*, *TaAGO7a*, *TaAGO8*, and *TaAGO9*), and two from the *TaRDR* family (*TaRDR2b* and *TaRDR2c*). Interestingly, the gene *TaDCL3a* from the DCL family is regulated by all four TF families which were assumed to possess high biological importance in regulating gene expression in wheat.

### 3.8. *cis*-Regulatory Elements (CREs) of the Predicted RNAi Genes

**Figure 8:**
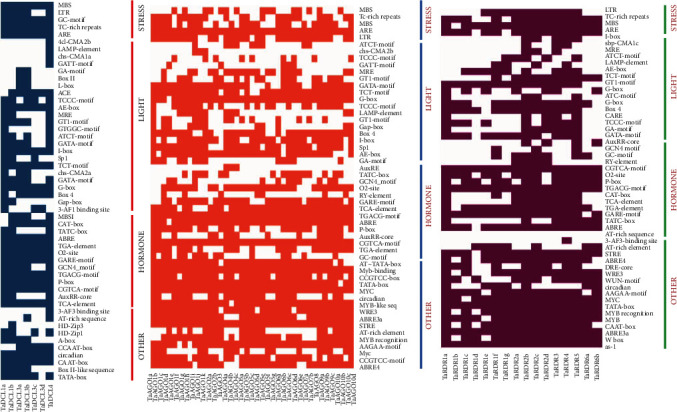
The *cis*-regulatory elements (CREs) for the predicted RNAi genes (*TaDCL*s, *TaAGO*s, and *TaRDR*s). The dark colour represents the existence of CREs corresponding to each of the predicted RNAi genes of wheat.

*cis*-Regulatory elements (CREs) consist of noncoding DNA motifs (5 ± 20 bp). They possess binding regions for TF and/or other regulatory molecules for triggering gene transcription [111]. They create defensive mechanisms in plants against different biotic and abiotic stresses [112] and take part in activities for development and physiological actions by controlling gene expression [111]. The identified CREs from the PlantCARE database were grouped for various actions such as being stress responsive, light responsive, and hormone responsive. It was observed that the maximum number of the *cis*-regulatory short sequences/motifs was found in the light response (LR) group followed by the hormone response group (Figure 8 and Data S5-S7). Plants' photosynthesis is connected to the light response that typically takes place in leaves. Photosynthesis is also the key physiological parameter in wheat like other plants that relate ultimately in many aspects to increasing the grain quality and crop productivity [113]. An increased rate of photosynthesis can utilize the solar radiation properly which leads to early flowering time because flowering signals are produced in leaves [104]. Therefore, the LR-related identified CREs are assumed to have direct links to high photosynthesis rates in wheat plant leaves. The LR-CREs such as the ATCT-motif, ATC-motif, Box-4, AE-box, G-box, I-box, GAT-motif, and GT1-motif were shared by most of the RNA silencing machinery genes in wheat [112, 114]. The TC-rich repeats (involved in defense and stress responsiveness [115]), MBS (MYB binding site) involved in drought inducibility (Chon et al., 2002), and LTR elements (CRE involved in low-temperature responsiveness) were commonly found as stress-responsive CREs among the predicted RNAi gene families.

A previous study reported that various plant hormones or phytohormones are essential for the plant's healthy growth and development [117]. Our analysis showed that 13–17 common CREs were associated with gene silencing activities for hormonal response in wheat. The ABRE *cis*-acting element is involved in abscisic acid responsiveness [118, 119]. The ABA (abscisic acid) is one of the key plant hormones that showed strong response and resistance by regulating many gene expressions against drought stress [120]. The expression of the many target proteins is regulated by ABA via ABA-responsive element (ABRE) binding protein/ABRE binding factor (AREB/ABF) transcription factors [120]. Both AuxRR and TGA are auxin-responsive growth hormones assumed to involve in regulating gene expression related to plant growth and development under extreme conditions in wheat like other important plants [112, 121, 122]. The role of these phytohormone auxins in developmental regulation is important against the cold condition in plants [123]. GC motif (enhancer-like element involved in anoxic-specific inducibility) [124, 125], GARE motif, P box, and TATC box are a type of gibberellin- (GA-) responsive element that are the important phytohormones responsible for various plant growth and development such as seed germination, shoot elongation, leaf expansion, flower development, and fruit senescence [126].

The O2-site element might be involved in zein metabolism regulation and circadian activities in wheat like *A. thaliana* and rice [112]. TCA element is another phytohormone-type CRE associated with salicylic acid responsiveness [127] that is found in wheat RNAi-related genes that were predicted to play a central role to control the plant development under biotic stresses [112]; the RY element found in wheat is assumed to be involved in seed-specific regulation such as to regulate gene expression during late embryogenesis and seed development [128]. The two CREs found in wheat such as MBSI (MYB binding site) and GCN4 motif were predicted to be involved in flavonoid biosynthetic gene regulation and endosperm expression [112]. Other than the role of hormonal responses in plants, these two CREs also contribute to cellular development in plants [112]. The CGTCA motif and TGACG motif shared by the wheat RNAi-related genes suggested that they are responsible for hormonal regulation specifically methyl jasmonate in plants [112]. They also help plants to survive against different environmental stresses [129]. CAT-box (*cis*-acting regulatory element related to meristem-specific activation) [112] were predicted as the hormone-responsive CREs in wheat (Figure 8 and Data S5-S7). Some previous studies also suggested that the five most important plant hormones: auxin, gibberellin, cytokinin, ethylene, and abscisic acid, act collectively or individually to affect plant growth and development under different biotic and abiotic stress conditions [112, 117, 130]. Hence, the above CREs associated with the *TaDCL*, *TaAGO*, and *TaRDR* gene families are known as the phytohormones and are assumed to be responsible for hormonal and stress responses, as well as cellular development in regulating wheat plant growth and development.

### 3.9. Expression Analysis of the Predicted RNAi Genes

**Figure 9:**
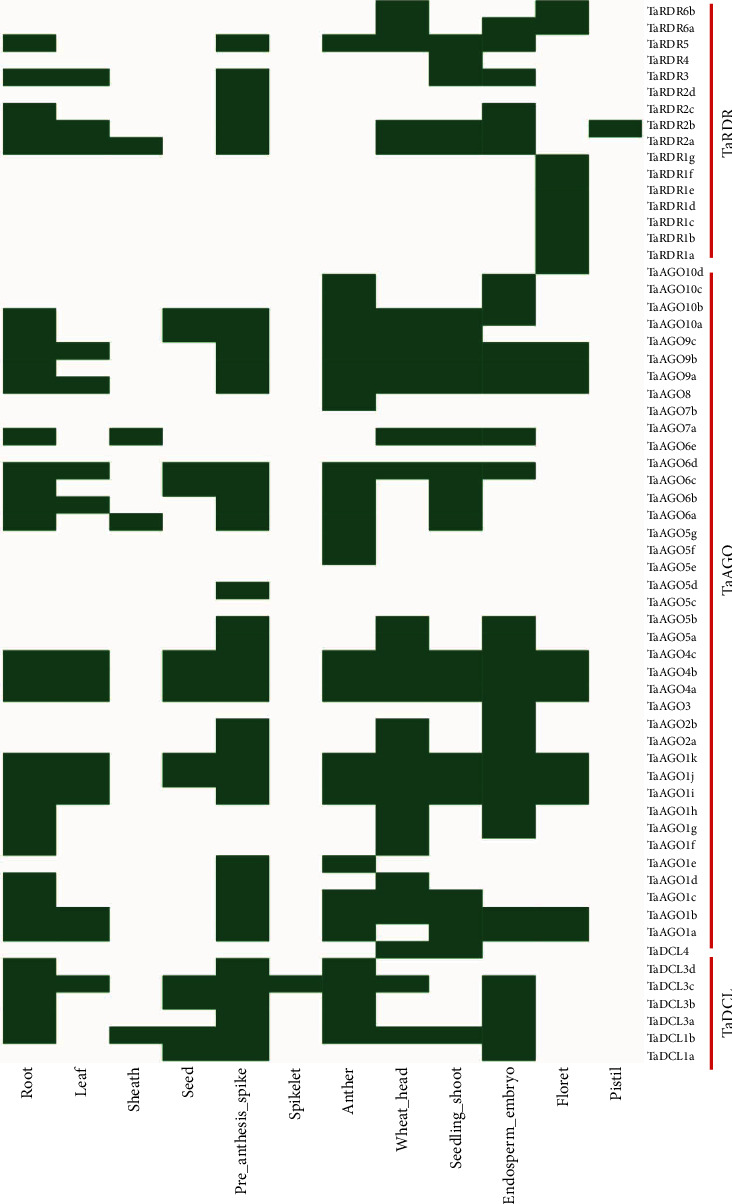
Expression analysis of the predicted RNAi genes by using an online database PlantGDB. The green colour represents the existence of expression of the corresponding genes in various tissues and organs of wheat.

To obtain further insights into the genomic information about gene expression at various organs or tissues in different conditions/environments, expressed sequence tag (EST) analysis was carried out for the identified 62 genes from PlantGDB. The analysis results showed that many RNA silencing machinery genes of the DCL, AGO, and RDR groups exhibited their expression in several important tissues and organs in wheat. Moreover, the expression study of the gene members of DCL, AGO, and RDR groups in several plant species was investigated, and thus, the analysis results reported that RNAi-dependent pathway genes showed significant expression eminence in the root, leaf, flower, seed, endosperm, spike, and other important organs or tissues ([2, 16, 20, 21, 26, 81]). It was clear in Figure 9 that almost all the members of *TaDCL*, *TaAGO*, and *TaRDR* gene families exhibited their expression at least in one tissue or organ while *TaAGO5c*, *TaAGO6e*, and *TaAOG7b* did not show any expression in any tissue or organ in wheat. Nearly 50% of the identified genes are expressed in the root, spike, anther, heads, shoots, and endosperm followed by the floret, leaf, and seed (Figure 9), which certainly implied that these tissues and organs provide a major contribution to improved wheat grain formation resulting in increased wheat yield. Among the 7 *TaDCL* gene members, *TaDCL4* showed expression in the root, head, and seedling shoot. All members of the *TaDCL3* group displayed expression in the root, spike, anther, and endosperm except *TaDCL3d* which exhibited endosperm-specific expression. On the other hand, *TaDCL1a* and *TaDCL1b* had no root-, leaf-, wheat head-/shoot-, and floret-/flower-specific expression, but importantly, they had only endosperm-embryo-, seed-, and spike-specific expression characteristics. It indicates that these two gene members may play roles in the formation of a healthy and sufficient number of grains in wheat. In rice, *OsDCL1b* and *OsDCL3b* exhibited panicle- and seed-specific expression whereas *SHO1*, *OsDCL1a*, *OsDCL2*, and *OsDCL3a* showed low expression in late seed development as well as displayed maximum expression in vegetative tissues such as young seedlings, leaf, root, and shoot apical meristem (SAM) [15]. Like rice, *AtDCL1*, *AtDCL2*, and *AtDCL3* also exhibited expression in all tissues linked to developmental stages such as in the leaf, root, flower, and seed but *AtDCL3* is only expressed in tissues related to flower [15].

Among the *TaAGO* and *TaRDR* gene families, *TaAGO1e*, *TaAGO3*, *TaAGO2a*/*2b*, *TaAGO10d*/*10c*, all 7 members of the *TaAGO5* subgroup, *TaRDR2d*, *TaRDR4*, and *TaRDR6a*/*6b* had no root-specific expression (Figure 9), though the *TaAGO2a*/*2b* genes expressed in endosperm, head, and spike implied that these two genes contribute to grain quality development. However, surprisingly, three members of the *TaAGO1* group (*TaAGO1i*/*j*/*k*), as well as all three members of the *TaAGO4* and *TaAGO9* groups, showed expression in all mentioned tissues and organs except pistil, spikelet, and sheath. This indicated that these 6 members of both the *TaAGO4* and *TaAGO9* groups might have roles in the growth and development of wheat. The gene members of the *TaAGO3* group had only endosperm-embryo-specific expression. The three gene members *TaAGO6a*/*6b*/*6c* had shoot anther-, spike-, and root-specific expression but the two members *TaAGO6c*/*6d* showed leaf-specific and the gene *TaAGO6d* showed only endosperm-specific expression. *TaAGO7a* also showed root-, head-/shoot-, and endosperm-specific expression. Among the four members of the *TaAGO10* gene group, the two genes *TaAGO10a*/*10b* displayed expression in all tissues and organs in wheat except the leaf, sheath, and floret. Previous experimental results reported that most of the AGO genes in rice (*OsAGO1a*, *1b*, *1c*, *1d*, *2*, *4a*, *4b*, *13*, *17*, *18*, and *OsPNH1*) expressed in vegetative and reproductive tissues/organs, viz., the leaf, root, panicle, inflorescence/meristem, seed, and seedling, and the remaining AGO genes had hardly any expression in the suggested tissues/organs [15]. On the other hand, all AGO genes in *A. thaliana* are expressed in the leaf, root, flower, silique, and seedling of which *AtAGO5*, *AtAGO7*, and *AtAGO9* showed no or little expression in the leaf and root and *AtAGO3* only expressed in silique [15]. Also, some earlier wet-lab studies reported that RNAi-related genes of the AGO family tend to show diverse expression intensity in the leaf, stem, and flower in *Brassica napus* and pepper (*Capsicum annuum*) [2, 22].

Furthermore, the EST analysis for all members of the *TaRDR1* gene group showed their expression in flowers but had no expression in any tissues and organs in wheat (Figure 9). It suggested that these genes tend to be responsible for expression in flowers like *TaRDR6a/6b*. A previous study on pepper (*Capsicum annuum*) reported that a large number of genes of the RDR gene family showed flower-specific expression [2]. In contrast, four members of the *TaRDR2* family did not provide any expression in flowers. The members of the *TaRDR2* gene group showed expression in all the cited tissues and organs with additional expression of *TaRDR2b* and *TaRDR2d* in the pistil and spike (Figure 9). A study on rice reported that the five RDR genes exhibited expression in vegetative and floral tissues while *OsRDR4* showed no expression except in SAM only [15]. Though *AtRDR1*, *AtRDR2*, and *AtRDR6* showed expression in the leaf, root, and flower in an early stage of seed formation and seedling, *AtRDR5* presented expression only in the root and increased expression in the late developmental stage of the seed [15].

It was observed from this *in silico* expression analysis of the 62 genes in wheat that genes expressed in the root and leaf are assumed to play a significant role in resisting abiotic stresses such as drought and waterlogging. Genes expressed in leaves may contribute to promoting the photosynthesis rate to produce sufficient energy for the wheat plants. Increased photosynthesis levels stimulate the flowering time of the crop because the respective signals are produced in leaves (Shanta). Seed- and endosperm-specific expressions of the RNAi-related genes imply that they may have an important role in maintaining quality seed formation.

**Figure 10:**
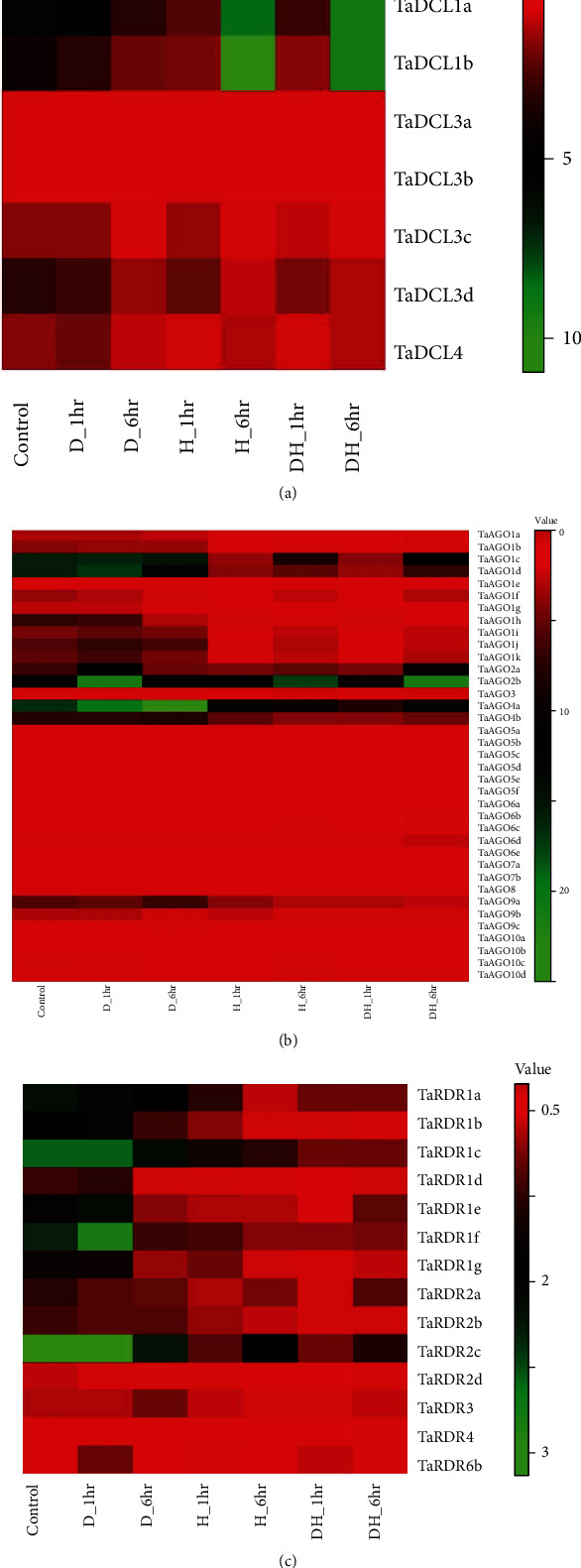
Heatmap displaying the expression pattern of (a) *TaDCL*, (b) *TaAGO*, and (c) *TaRDR* genes under drought, heat, a mixture of drought-heat stress, and control conditions. Relative expression levels were collected from the WheatExp database, and heatmaps were created using the Heatmapper web tool. The colour scale representing signal values is displayed above the heat map. Green represents the high-level and red indicated the low-level expression or transcript abundance.

The expression analysis was also carried out for all the putative RNAi genes from the WheatExp database under drought and heat stress conditions as well (Figure 10). The analysis showed that among the *TaDCL*s, only *TaDCL1a* and *TaDCL1b* exhibited high expression (fold > 5) during drought conditions and showed maximum expression at 6-hour heat (H_6hr) conditions (fold > 10) (Figure 10(a)). Other than these two DCLs, the *TaDCL3b*, *TaDCL3d*, and *TaDCL4* also exhibited drought and heat stress tolerance expression (1 < fold < 3). On the other hand, the 5 *TaAGO* genes such as *TaAGO1c*, *TaAGO1d*, *TaAGO2a*, *TaAGO2b*, and *TaAGO4a* showed high expression at drought stress (fold > 10) (Figure 10(b)) whereas in heat and combined heat-drought conditions, only the two genes *TaAGO2b* and *TaAGO4a* showed high expression (fold > 10). Though the gene *TaAGO1c* displayed high expression (fold > 10) at D_6hr and DH_6hr, *TaAGO2a* showed a high expression level (fold > 10) only at DH_6hr (Figure 10(b)). The two genes *TaAGO4b* and *TaAGO9a* were predicted to show some high expression (5 < fold < 10) during drought conditions (D_1hr and D_6hr). Among the RDR genes, the *TaRDR2c* only exhibited high expression (fold > 2) under all conditions: drought, heat, and combined drought-heat conditions (Figure 10(c)). Out of the remaining RDR genes, all members of the *TaRDR1* group except *TaRDR1d* showed high expression (fold > 1.5) under drought conditions (D_1hr and D_6hr) and only *TaRDR1a* and *TaRDR1c* exhibited some higher expression level during heat stress conditions (Figure 10(c)).

## 4. Conclusion

Integrated bioinformatic analyses were carried out for the identification and characterization of wheat RNAi gene families (*DCL*, *AGO*, and *RDR*) highlighting their regulatory components. Results showed that the wheat genome possessed 7 DCL-, 39 AGO-, and 16 RDR RNAi-related genes. The initial analysis provided some standard genomic and physicochemical info on the identified genes. The phylogenetic analysis showed that all members of the three groups maintained their evolutionary relationships similar to their rice and *Arabidopsis* homologs. The first group of *TaDCL*, *TaAGO*, and *TaRDR* possessed multiple copies of genes higher than those of their rice and *Arabidopsis* counterparts. Conserved functional domain and motif structure analyses showed that the genes also contained consistent functional domains and motif structures similar to those of the rice and *Arabidopsis* RNAi-related genes. The 3D protein structures and lower values of the Ka/Ks ratio implied that the protein sequences maintain some particular functions evolutionarily encoded in the protein sequences. The most important regulatory relationship networks among the popular TF families and the RNAi-related genes were generated and ERF was identified as the most potential TF family. The predicted CREs were related to mostly light, stress, and hormone responses. The CRE and expression analyses hence showed that the predicted genes had diversifying participation in plant growth, development, and abiotic stress tolerance maintaining the grain quality and increasing crop production in wheat. The results of this study would provide an important indication of evolutionary resemblances of DCL, AGO, and RDR genes in wheat with their rice and *Arabidopsis* counterparts. Analysis results would therefore provide valuable sources for further biological and molecular justification and implementation to draw a more specific conclusion regarding any particular gene(s) and its domain of activity against different biotic and abiotic stresses as well as growth and development in wheat for more improvement of this potential cereal crop.

## Figures and Tables

**Table 1:** Basic genomic information about gene families *TaDCL*, *TaAGO*, and *TaRDR* and properties of the encoded proteins in wheat (*T. aestivum*).

Gene no.	Gene name	Accession number	Genomic location	Gene length (bp)	Protein				Type
					No. of introns	Molecular weight (D.a)	Protein length (a.a.)	pI	
DCL									
1	*TaDCL1a*	Traes_4BL_B3A1B8342.2	ta_iwgsc_4bl_v1_6994694:2688..13103	10416	18	195505.38	1756	6.43	DCL1
2	*TaDCL1b*	Traes_5AL_72A7552B9.2	ta_iwgsc_5al_v1_2752914:2855..12171	9317	17	187281.30	1674	6.19	DCL1
3	*TaDCL3a*	Traes_1AL_E7144546E.1	ta_iwgsc_1al_v2_3918617:7862..20042	12181	25	184016.48	1627	6.39	DCL3
4	*TaDCL3b*	Traes_1DL_C646B6990.1	ta_iwgsc_1dl_v1_2224660:33..11929	11897	22	169269.68	1494	6.46	DCL3
5	*TaDCL3c*	Traes_3AL_562D6614F.1	ta_iwgsc_3al_v1_4401047:3091..12058	8968	23	177350.72	1586	6.04	DCL3
6	*TaDCL3d*	Traes_3DL_2DC78B18A.1	ta_iwgsc_3dl_v1_6937552:192..8538	8347	23	177414.90	1586	6.06	DCL3
7	*TaDCL4*	Traes_2DL_E96DCDCB4.2	ta_iwgsc_2dl_v1_9883052:5868..20610	14743	18	157968.69	1392	5.92	DCL4
AGO									
1	*TaAGO1a*	Traes_2AL_2512A7F91.1	ta_iwgsc_2al_v1_6428016:16577..22920	6344	21	123940.53	1117	9.48	AGO1
2	*TaAGO1b*	Traes_2BL_93099ACF4.1	ta_iwgsc_6al_v1_5742333:938..12517	11580	16	72763.47	645	9.21	AGO1
3	*TaAGO1c*	Traes_6AL_616161AAB.1	ta_iwgsc_6al_v1_5742333:938..12517	11580	21	108532.11	978	9.48	AGO1
4	*TaAGO1d*	Traes_6DL_58620B158.2	ta_iwgsc_6dl_v1_3210843:3..6281	6279	21	101586.27	910	9.35	AGO1
5	*TaAGO1e*	Traes_7DL_C255A109C.1	ta_iwgsc_7dl_v1_3396214:4962..9930	4969	14	77484.04	721	9.22	AGO1
6	*TaAGO1f*	Traes_6BL_9CFA54D4A.1	ta_iwgsc_6bl_v1_4398549:8497..16669	8173	21	119364.74	1068	9.56	AGO1
7	*TaAGO1g*	Traes_6AL_317133B3F.2	ta_iwgsc_6al_v1_5770608:7589..20031	12443	22	120920.18	1085	9.60	AGO1
8	*TaAGO1h*	Traes_6DL_804FB7F75.1	ta_iwgsc_6dl_v1_3318900:4215..12555	8341	21	119065.35	1067	9.58	AGO1
9	*TaAGO1i*	Traes_7AS_56569A5AC.2	ta_iwgsc_7as_v1_4062808:1122..8394	7273	21	133941.63	1210	9.41	AGO1
10	*TaAGO1j*	Traes_7DS_4D01B6175.1	ta_iwgsc_7ds_v1_3925271:1281..7139	5859	21	97926.28	868	9.31	AGO1
11	*TaAGO1k*	Traes_4AL_A118C6C84.2	ta_iwgsc_4al_v2_7127490:1302..7644	6343	21	131032.87	1189	9.51	AGO1
12	*TaAGO2a*	Traes_2AL_DFE4C65F6.2	ta_iwgsc_2al_v1_6435863:9..4673	4665	2	104716.17	952	9.23	AGO2
13	*TaAGO2b*	Traes_2BL_7713B3533.2	ta_iwgsc_2bl_v1_8046272:2..4842	4841	2	94598.13	845	9.17	AGO2
14	*TaAGO3*	Traes_2DL_A77212060.2	ta_iwgsc_2dl_v1_9902460:2552..5785	3234	2	95781.21	849	8.54	AGO3
15	*TaAGO4a*	Traes_3AS_8EE711E2C.2	ta_iwgsc_3as_v1_3376626:7020..14138	7119	21	111230.93	999	8.88	AGO4
16	*TaAGO4b*	Traes_3DS_57EA31670.1	ta_iwgsc_3ds_v1_2601767:2..5053	5052	17	88926.36	799	9.22	AGO4
17	*TaAGO4c*	Traes_3B_F4E4667F8.1	ta_iwgsc_3b_v1_10588096:6226..11781	5556	20	99622.27	918	9.21	AGO4
18	*TaAGO5a*	Traes_2BS_8368F6B5D.1	ta_iwgsc_2bs_v1_5155056:10..6355	6346	21	91375.57	815	9.02	AGO4
19	*TaAGO5b*	Traes_2DS_4CC8FD7E3.1	ta_iwgsc_2ds_v1_5380297:2..6639	6638	21	94300.29	838	9.10	AGO5
20	*TaAGO5c*	Traes_5BL_F505BF164.1	ta_iwgsc_5bl_v1_10819703:15714..21283	5570	19	82474.57	732	8.95	AGO5
21	*TaAGO5d*	Traes_4AL_7CC35DF1D.2	ta_iwgsc_4al_v2_7146652:9555..15647	6093	21	98771.25	875	9.18	AGO5
22	*TaAGO5e*	Traes_4DS_88D2821C6.2	ta_iwgsc_4ds_v1_2318247:117..6407	6291	21	103082.25	916	9.22	AGO5
23	*TaAGO5f*	Traes_3AS_3F8424E4E.1	ta_iwgsc_3as_v1_1779497:2..5110	5109	20	93220.12	832	9.18	AGO5
24	*TaAGO5g*	Traes_3B_CA99AB66C.1	ta_iwgsc_3b_v1_9317732:662..5865	5204	20	94133.21	841	9.25	AGO5
25	*TaAGO6a*	Traes_1BL_05F7B7DFA.1	ta_iwgsc_1bl_v1_2609948:748..7252	6505	19	100043.38	893	6.99	AGO5
26	*TaAGO6b*	Traes_5AL_07EFD5712.1	ta_iwgsc_5al_v1_2801172:3925..11424	7500	21	98228.56	883	9.31	AGO6
27	*TaAGO6c*	Traes_5DL_672EE3605.1	ta_iwgsc_5dl_v1_4504514:5254..15341	10088	21	98062.91	882	9.31	AGO6
28	*TaAGO6d*	Traes_5BL_F611D65E0.1	ta_iwgsc_5bl_v1_10861763:7394..17699	10306	21	98241.09	883	9.30	AGO6
29	*TaAGO6e*	Traes_7AL_D88450A3C.2	ta_iwgsc_7al_v1_944917:567..5236	4670	17	86268.90	768	9.09	AGO6
30	*TaAGO7a*	Traes_2AL_3F3117458.1	ta_iwgsc_2al_v1_6334464:10651..14850	4200	2	105787.59	934	9.23	AGO7
31	*TaAGO7b*	Traes_2BL_24111235C.1	ta_iwgsc_2bl_v1_7955795:2637..7172	4536	2	115033.19	1023	9.33	AGO7
32	*TaAGO8*	Traes_7AL_1BAB53DCE.1	ta_iwgsc_7al_v1_4478819:3359..8183	4825	21	101590.86	901	9.11	AGO8
33	*TaAGO9a*	Traes_1AL_095416BC0.1	ta_iwgsc_1al_v2_3876661:1149..9699	8551	20	102455.28	925	9.09	AGO9
34	*TaAGO9b*	Traes_1BL_7C037D478.2	ta_iwgsc_1bl_v1_3899789:1061..10207	9147	21	102872.66	927	9.06	AGO9
35	*TaAGO9c*	Traes_1DL_64B330BBB.2	ta_iwgsc_1dl_v1_2275968:1227..11215	9989	21	102361.16	922	9.06	AGO9
36	*TaAGO10a*	Traes_6AS_FBB2AFAAB.1	ta_iwgsc_6as_v1_4364847:5932..12979	7048	20	105777.22	948	9.37	AGO10
37	*TaAGO10b*	Traes_6DS_9DD64BD48.1	ta_iwgsc_6ds_v1_2082993:2072..10005	7934	20	99080.09	883	9.95	AGO10
38	*TaAGO10c*	Traes_7AL_96766587F.2	ta_iwgsc_7al_v1_4543530:3452..7823	4372	15	65968.92	583	8.88	AGO10
39.	*TaAGO10d*	Traes_7DL_C538856D4.1	ta_iwgsc_7dl_v1_3364674:1..5438	5438	18	74927.28	664	8.95	AGO10
*RDR*									
1	*TaRDR1a*	Traes_6DL_4B89E8742.2	ta_iwgsc_6dl_v1_3272251:691..6878	6188	3	127488.70	1120	6.98	RDR1
2	*TaRDR1b*	Traes_6BL_78BEF51DD.1	ta_iwgsc_6bl_v1_4353480:4946..8011	3066	2	82713.43	727	6.82	RDR1
3	*TaRDR1c*	Traes_6AL_393C6B853.1	ta_iwgsc_6al_v1_5823227:4526..9827	5302	3	127483.82	1119	7.15	RDR1
4	*TaRDR1d*	Traes_6BL_0A9D15EDC.2	ta_iwgsc_6bl_v1_4254864:3..1709	1707	1	54258.16	484	8.42	RDR1
5	*TaRDR1e*	Traes_6BL_0BB5C493D.1	ta_iwgsc_6bl_v1_4369818:3771..6377	2607	2	38289.35	333	5.40	RDR1
6	*TaRDR1f*	Traes_6AL_13BC97E04.1	ta_iwgsc_6al_v1_5769298:833..7192	6360	3	127303.48	1116	8.53	RDR1
7	*TaRDR1g*	Traes_6BL_DF680C2AF.1	ta_iwgsc_6bl_v1_4369819:1195..5786	4592	4	108562.11	947	7.34	RDR1
8	*TaRDR2a*	Traes_2DL_6DB81005E.1	ta_iwgsc_2dl_v1_9891666:1..7150	7150	3	82516.60	732	6.38	RDR2
9	*TaRDR2b*	Traes_4AS_8D6311711.1	ta_iwgsc_4as_v2_5940771:10843..26735	15893	4	126947.12	1127	6.60	RDR2
10	*TaRDR2c*	Traes_4DL_2E9CE89D9.2	ta_iwgsc_4dl_v3_14391157:7408..18499	11092	4	130417.41	1156	8.15	RDR2
11	*TaRDR2d*	Traes_4DL_A54C80661.1	ta_iwgsc_4dl_v3_14383226:1730..7149	5420	2	97124.16	869	6.82	RDR2
12	*TaRDR3*	Traes_7BL_8CEC8F99B.2	ta_iwgsc_7bl_v1_6687438:7..10355	10349	13	70361.58	616	6.20	RDR3
13	*TaRDR4*	Traes_3AS_F27BB108C.2	ta_iwgsc_3as_v1_3345716:2930..10167	7238	6	50505.60	436	5.75	RDR4
14	*TaRDR5*	Traes_3B_2C6DB84FB.2	ta_iwgsc_3b_v1_10500163:2098..12737	10640	11	63011.89	560	6.84	RDR5
15	*TaRDR6a*	Traes_3B_DC77B5E89.1	ta_iwgsc_3b_v1_10414255:3..4337	4335	1	104445.56	923	7.29	RDR6
16	*TaRDR6b*	Traes_3DL_F32B49981.1	ta_iwgsc_3dl_v1_6945798:1..3009	3009	1	60535.30	543	6.71	RDR6

Genomic info such as gene names, accession number, genomic/chromosomal location, genome length, and protein length was collected from the Phytozome database (https://phytozome.jgi.doe.gov/pz/portal.html), and the molecular weight and pI values were predicted by the ExPASy ComputepI/Mwtool (http://au.expasy.org/tools /pi_tool.html). Molecular weights are in Daltons and “aa” means amino acid.

**Table 2:** Comparison of Argonaute proteins with missing catalytic residue(s) present in the PIWI domain of wheat, rice^b^, and *Arabidopsis*^b^.

	*Wheat*		Rice		*Arabidopsis thaliana*	
	Argonaute	Motif^a^	Argonaute	Motif^b^	Argonaute	Motif^b^
1	*TaAGO1a*	DDH/H	*OsAGO1*	DDH/P	*AGO2*	DDD/H
2	*TaAGO1b*	DDH/H	*OsAGO2*	DDD/H	*AGO3*	DDD/H
3	*TaAGO1c*	DDH/H	*OsAGO3*	DDD/H	*AGO4*	DDH/S
4	*TaAGO1d*	DDH/H	*OsAGO4a*	DDH/P	*AGO6*	DDH/P
5	*TaAGO1e*	DD-/H	*OsAGO4b*	DDH/P	*AGO9*	DDH/R
6	*TaAGO1f*	DDH/R	*OsAGO11*	GDH/H		
7	*TaAGO1g*	DDH/R	*OsAGO13*	-DH/H		
8	*TaAGO1h*	DDH/R	*OsAGO15*	DDH/P		
9	*TaAGO1i*	DDH/H	*OsAGO16*	DDH/P		
10	*TaAGO1j*	DDH/H	*OsAGO17*	HDR/C		
11	*TaAGO1k*	DDH/H	*OsAGO18*	DDH/S		
12	*TaAGO2a*	DDD/H				
13	*TaAGO2b*	DDD/H				
14	*TaAGO3*	DDD/H				
15	*TaAGO4a*	DDH/P				
16	*TaAGO4b*	DD-/P				
17	*TaAGO4c*	DDH/P				
18	*TaAGO5a*	DDH/P				
19	*TaAGO5b*	DDH/P				
20	*TaAGO5c*	DDH/H				
21	*TaAGO5d*	DDH/H				
22	*TaAGO5e*	DDH/P				
23	*TaAGO5f*	DDH/H				
24	*TaAGO5g*	DDH/H				
25	*TaAGO6a*	DD-/P				
26	*TaAGO6b*	DDH/P				
27	*TaAGO6c*	DDH/P				
28	*TaAGO6d*	DDH/P				
29	*TaAGO6e*	DD-/P				
30	*TaAGO7a*	DDH/H				
31	*TaAGO7b*	DDH/H				
32	*TaAGO8*	YDH/P				
33	*TaAGO9a*	DDH/P				
34	*TaAGO9b*	DDH/P				
35	*TaAGO9c*	DDH/P				
36	*TaAGO10a*	DDH/Y				
37	*TaAGO10b*	DDH/Y				
38	*TaAGO10c*	DDH/H				
39	*TaAGO10d*	DDH/H				

^a^Motifs are corresponding to conserved D760, D845, **and** H986/H798 of Arabidopsis AGO1; D (aspartate), H (Histidine), K (Lysine), A (Alanine), P (Proline), Q (Glutamine), G (Glycine), R (Arginine), C (Cysteine), S (Serine); –: the missing catalytic residue. ^b^Reviewed in Kapoor et al. [15].

## Data Availability

All data generated or analyzed during this study were provided as supplementary files.

## Supplementary Materials

Supplementary 1 Table S1: list of predicted conserved functional domains of the DCL, AGO, and RDR proteins in wheat using Pfam, SMART, and NCBI-CDD. Table S2: list of the number of alpha-helices, beta-strands at the 3D structure level, and Ka/Ks ratios of the RNAi-dependent pathway proteins in wheat. Table S3: percentage of three groups of RNA silencing genes involved in the different cellular locations in wheat. Table S4: distribution of TF families regulating RNAi-based genes in wheat.

Supplementary 2 Data S1: protein sequences of the identified DCL genes in wheat. Data S2: protein sequences of the identified AGO genes in wheat. Data S3: protein sequences of the identified RDR genes in wheat. Data S4: list of transcript factors and their families regulating the predicted RNAi-based genes. Data S5: list of *cis*-regulatory elements associated with the TaDCL protein families. Data S6: list of *cis*-regulatory elements associated with the TaAGO protein families. Data S7: list of *cis-*regulatory elements associated with the TaRDR protein families.

Supplementary 3 Figure S1: alignment of catalytic regions in RdRp domains of RDR proteins of wheat, rice, and *A. thaliana*. Figure S2: discovery of 20 conserved motif structures. Figure S3A-C: three-dimensional protein structure of the (A) TaDCL, (B) TaAGO, and (C) TaRDR proteins of wheat. Figure S4: regulatory gene network among the TF families and the predicted RNAi-based genes in wheat. Figure S5: distribution of TF families corresponding to each RNAi gene member. Figure S6: RNAi gene-mediated subnetworks with different TF families. (A) ERF, (B) MIKC-MADS, (C) C2H2, (D) BBR-BPC, (E) MYB, and (F) Dof TF family.
