# Supplementary material for: Comprehensive In Silico Analysis of RNA Silencing-Related Genes and Their Regulatory Elements in Wheat (Triticum aestivum L.)
Source: Biomed Res Int. 2022 Sep 19;2022:4955209. doi: 10.1155/2022/4955209 (PMC9513535; doi:10.1155/2022/4955209)
Supplement: Supplementary 1 — Table S1: list of predicted conserved functional domains of the DCL, AGO, and RDR proteins in wheat using Pfam, SMART, and NCBI-CDD. Table S2: list of the number of alpha-helices, beta-strands at the 3D structure level, and Ka/Ks ratios of the RNAi-dependent pathway proteins in wheat. Table S3: percentage of three groups of RNA silencing genes involved in the different cellular locations in wheat. Table S4: distribution of TF families regulating RNAi-based genes in wheat. [file 4955209.f1.doc]

**Table S1. List of predicted conserved functional domains of the DCL, AGO, and RDR proteins in wheat using Pfam, SMART, and NCBI-CDD.**

| Gene  No. | | Gene  Name | | Accession Number | | Domains | | | | | |
| --- | --- | --- | --- | --- | --- | --- | --- | --- | --- | --- | --- |
|  |  | |  | | Pfam | | SMART | | NCBI-CDD | |  |
| DCL | | | | | | | | | | | |
| 1. | | TaDCL1a | | Traes_4BL_B3A1B8342.2 | | Ribonuclease_3,Ribonuclease_3,  PAZ,Dicer_dimer,Helicase_C,DND1_DSRM,ResIII,dsrm | | DEXDc,HELICc,PAZ,RIBOc,RIBOc,DSRM,DSRM | | DEXHc_dicer, SF2_C_dicer(C-terminal domain/HelicC), PAZ super family, RIBOc, RIBOc, Dicer_dimer, DSRM_SF super family, DSRM_SF super family | |
| 2. | | TaDCL1b | | Traes_5AL_72A7552B9.2 | | Ribonuclease_3,Ribonuclease_3,  PAZ,Dicer_dimer,Helicase_C,DND1_DSRM, dsrm, ResII,, DUF283 | | DEXDc,HELICc,PAZ,RIBOc,RIBOc,DSRM,DSRM | | DEXHc_dicer, PAZ_CAF_like, SF2_C_dicer(C-terminal domain/HelicC), Rnc, DSRM_DCL_plant, RIBOc, Dicer_dimer | |
| 3. | | TaDCL3a | | Traes_1AL_E7144546E.1 | | Ribonuclease_3,Ribonuclease_3,  Dicer_dimer,Helicase_C,DEAD,PAZ,dsrm, DND1_DSRM | | DEXDc,HELICc,PAZ,RIBOc,RIBOc,DSRM,DSRM | | DEXHc_dicer, MPH1 super family(ERCC4-related helicase), RIBOc, Dicer_dimer, RIBOc, DSRM_SF super family | |
| 4. | | TaDCL3b | | Traes_1DL_C646B6990.1 | | Ribonuclease_3,Ribonuclease_3,  Dicer_dimer,Helicase_C,PAZ,dsrm, DND1_DSRM | | HELICc,PAZ,RIBOc,RIBOc,DSRM,DSRM | | RIBOc, DEAD-like_helicase_N super family, DEAD-like_helicase_C super family(also called Helicase-C), Dicer_dimer, RIBOc, PAZ super family, DSRM_SF super family | |
| 5. | | TaDCL3c | | Traes_3AL_562D6614F.1 | | Ribonuclease_3,Ribonuclease_3,  Dicer_dimer,Helicase_C,PAZ,ResIII, dsrm, DND1_DSRM | | DEXDc, HELICc, DSRM, PAZ, RIBOc, RIBOc, DSRM | | DEXHc_dicer, SF2_C_dicer (C-terminal domain/HelicC), Dicer_dimer, PAZ super family , RIBOc RIBOc, , DSRM_SF super family, DSRM_SF super family | |
| 6. | | TaDCL3d | | Traes_3DL_2DC78B18A.1 | | Ribonuclease_3,Ribonuclease_3,  Dicer_dimer,Helicase_C,PAZ,ResIII,dsrm | | DEXDc,HELICc,DSRM,PAZ,RIBOc,RIBOc,DSRM, DSRM | | DEXHc_dicer, SF2_C_dicer (C-terminal domain/HelicC), PAZ super family ,RIBOc , RIBOc, Dicer_dimer, DSRM_SF super family, DSRM_SF super family | |
| 7. | | TaDCL4 | | Traes_2DL_E96DCDCB4.2 | | Ribonuclease_3,Ribonuclease_3,  Dicer_dimer,Helicase_C,DND1_DSRM,PAZ,dsrm | | HELICc,PAZ,RIBOc,RIBOc,DSRM | | DEAD-like_helicase_C super family(also called Helicase-C), Rnc, PAZ super family , Dicer_dimer, RIBOc, DSRM_SF super family, DSRM_SF super | |
| AGO | | | | | | | | | | | |
| 1 | | TaAGO1a | | Traes_2AL_2512A7F91.1 | | Piwi,Gly-rich_Ago1,ArgoN,PAZ,ArgoL1,ArgoL2,ArgoMid | | DUF1785,PAZ,Piwi | | Gly-rich-Ago, ArgoN, ArgoL1, PAZ-argonaute, PIWI | |
| 2. | | TaAGO1b | | Traes_2BL_93099ACF4.1 | | Piwi,PAZ,ArgoL2 | | PAZ,Piwi | | PAZ, PIWI | |
| 3. | | TaAGO1c | | Traes_6AL_616161AAB.1 | | Piwi,ArgoN,PAZ,ArgoL1,ArgoL2,ArgoMid | | DUF1785,PAZ,Piwi | | ArgoN, ArgoL1, PAZ, PIWI | |
| 4. | | TaAGO1d | | Traes_6DL_58620B158.2 | | Piwi,ArgoN,PAZ,ArgoL1,ArgoL2,ArgoMid | | DUF1785,PAZ,Piwi | | ArgoN, ArgoL1, PAZ, PIWI | |
| 5. | | TaAGO1e | | Traes_7DL_C255A109C.1 | | Piwi,ArgoN,PAZ,ArgoL1,ArgoMid | | DUF1785,PAZ,Piwi | | ArgoN, ArgoL1, PAZ, PIWI | |
| 6. | | TaAGO1f | | Traes_6BL_9CFA54D4A.1 | | Piwi,Gly-rich_Ago1,ArgoN,PAZ,ArgoL1,ArgoL2,ArgoMid | | DUF1785,PAZ,Piwi | | Gly-rich-Ago, ArgoN, ArgoL1, PAZ ,PIWI | |
| 7. | | TaAGO1g | | Traes_6AL_317133B3F.2 | | Piwi,Gly-rich_Ago1,ArgoN,PAZ,ArgoL1,ArgoL2,ArgoMid | | DUF1785,PAZ,Piwi | | Gly-rich-Ago, ArgoN, ArgoL1, PAZ, PIWI | |
| 8. | | TaAGO1h | | Traes_6DL_804FB7F75.1 | | Piwi,Gly-rich_Ago1,ArgoN,PAZ,ArgoL1,ArgoL2,ArgoMid | | DUF1785,PAZ,Piwi | | Gly-rich-Ago, ArgoN, ArgoL1, PAZ, PIWI | |
| 9. | | TaAGO1i | | Traes_7AS_56569A5AC.2 | | Piwi,Gly-rich_Ago1,ArgoN,PAZ,ArgoL1,ArgoL2,ArgoMid | | DUF1785,PAZ,Piwi | | Gly-rich-Ago, ArgoN, ArgoL1, PAZ, PIWI | |
| 10. | | TaAGO1j | | Traes_7DS_4D01B6175.1 | | Piwi,ArgoN,PAZ,ArgoL1,ArgoL2,ArgoMid | | DUF1785,PAZ,Piwi | | ArgoN, ArgoL1, PIWI | |
| 11. | | TaAGO1k | | Traes_4AL_A118C6C84.2 | | Piwi,ArgoN,PAZ,ArgoL1,ArgoL2,ArgoMid | | DUF1785,PAZ,Piwi | | Gly-rich-Ago | |
| 12. | | TaAGO2a | | Traes_2AL_DFE4C65F6.2 | | Piwi,PAZ,ArgoN,ArgoL1,ArgoL2,ArgoMid | | DUF1785,PAZ,Piwi | | ArgoN, ArgoL1, PAZ, PIWI | |
| 13. | | TaAGO2b | | Traes_2BL_7713B3533.2 | | Piwi,PAZ,ArgoN,ArgoL2,ArgoL1,ArgoMid | | DUF1785,PAZ,Piwi | | ArgoN, ArgoL1, PAZ, PIWI | |
| 14. | | TaAGO3 | | Traes_2DL_A77212060.2 | | Piwi,PAZ | | DUF1785,PAZ,Piwi | | ArgoL1, PAZ, ArgoN ,PIWI | |
| 15. | | TaAGO4a | | Traes_3AS_8EE711E2C.2 | | Piwi,ArgoN,PAZ,ArgoL1,ArgoL2 | | DUF1785,Piwi | | ArgoN, ArgoL1, PAZ, PIWI | |
| 16. | | TaAGO4b | | Traes_3DS_57EA31670.1 | | Piwi,ArgoN,PAZ,ArgoL1,ArgoL2 | | DUF1785,Piwi | | ArgoN, ArgoL1,PAZ, PIWI | |
| 17. | | TaAGO4c | | Traes_3B_F4E4667F8.1 | | Piwi,ArgoN,PAZ,ArgoL1,ArgoL2 | | DUF1785,Piwi | | ArgoN, ArgoL1, PAZ, PIWI | |
| 18. | | TaAGO5a | | Traes_2BS_8368F6B5D.1 | | Piwi,ArgoN,PAZ,ArgoL1,ArgoL2,ArgoMid | | DUF1785,PAZ,Piwi | | ArgoN, ArgoL1, PAZ, PIWI | |
| 19. | | TaAGO5b | | Traes_2DS_4CC8FD7E3.1 | | Piwi,ArgoN,PAZ,ArgoL1,ArgoL2,ArgoMid | | DUF1785,PAZ,Piwi | | ArgoN, ArgoL1, PAZ, PIWI | |
| 20. | | TaAGO5c | | Traes_5BL_F505BF164.1 | | Piwi,PAZ,ArgoL1,ArgoL2,ArgoMid | | DUF1785,PAZ,Piwi | | ArgoL1, PAZ, PIWI | |
| 21. | | TaAGO5d | | Traes_4AL_7CC35DF1D.2 | | Piwi,ArgoN,PAZ,ArgoL1,ArgoMid,ArgoL2 | | DUF1785,PAZ,Piwi | | ArgoN, ArgoL1, PAZ, PIWI | |
| 22. | | TaAGO5e | | Traes_4DS_88D2821C6.2 | | Piwi,ArgoN,PAZ,ArgoL1,ArgoMid,ArgoL2 | | DUF1785,PAZ,Piwi | | ArgoN, ArgoL1, PAZ, PIWI | |
| 23. | | TaAGO5f | | Traes_3AS_3F8424E4E.1 | | Piwi,ArgoN,PAZ,ArgoL1,ArgoMid,ArgoL2 | | DUF1785,PAZ,Piwi | | ArgoN, ArgoL1, PAZ, PIWI | |
| 24. | | TaAGO5g | | Traes_3B_CA99AB66C.1 | | Piwi,ArgoN,PAZ,ArgoL1,ArgoL2,ArgoMid | | DUF1785,PAZ,Piwi | | ArgoN, ArgoL1, PAZ, PIWI | |
| 25. | | TaAGO6a | | Traes_1BL_05F7B7DFA.1 | | Piwi,ArgoN,PAZ,ArgoL1,ArgoL2 | | DUF1785,PAZ,Piwi | | ArgoL1, PAZ, PIWI | |
| 26. | | TaAGO6b | | Traes_5AL_07EFD5712.1 | | Piwi,ArgoN,PAZ,ArgoL2,ArgoL1 | | DUF1785,PAZ,Piwi | | ArgoN, ArgoL1, PAZ, PIWI | |
| 27. | | TaAGO6c | | Traes_5DL_672EE3605.1 | | Piwi,ArgoN,PAZ,ArgoL2,ArgoL1 | | DUF1785,PAZ,Piwi | | ArgoN, ArgoL1, PAZ, PIWI | |
| 28. | | TaAGO6d | | Traes_5BL_F611D65E0.1 | | Piwi,ArgoN,PAZ,ArgoL2,ArgoL1 | | DUF1785,PAZ,Piwi | | ArgoN, ArgoL1, PAZ, PIWI | |
| 29. | | TaAGO6e | | Traes_7AL_D88450A3C.2 | | Piwi,PAZ,ArgoN,ArgoL2,ArgoL1 | | DUF1785,Piwi | | ArgoN, ArgoL1, PAZ, PIWI | |
| 30. | | TaAGO7a | | Traes_2AL_3F3117458.1 | | Piwi,ArgoN,PAZ,ArgoL1,ArgoL2 | | DUF1785,PAZ,Piwi | | ArgoN, ArgoL1, PAZ, PIWI | |
| 31. | | TaAGO7b | | Traes_2BL_24111235C.1 | | Piwi,ArgoN,PAZ,ArgoL1,ArgoL2 | | DUF1785,PAZ,Piwi | | ArgoN, ArgoL1, PAZ | |
| 32. | | TaAGO8 | | Traes_7AL_1BAB53DCE.1 | | Piwi,ArgoN,PAZ,ArgoL1,ArgoL2,ArgoMid | | DUF1785,PAZ,Piwi | | PIWI | |
| 33. | | TaAGO9a | | Traes_1AL_095416BC0.1 | | Piwi,ArgoN,PAZ,ArgoL1,ArgoL2 | | DUF1785,Piwi | | ArgoN, ArgoL1, PAZ, PIWI | |
| 34. | | TaAGO9b | | Traes_1BL_7C037D478.2 | | Piwi,ArgoN,PAZ,ArgoL1,ArgoL2 | | DUF1785,Piwi | | ArgoN, ArgoL1, PAZ, PIWI | |
| 35. | | TaAGO9c | | Traes_1DL_64B330BBB.2 | | Piwi,ArgoN,PAZ,ArgoL1,ArgoL2 | | DUF1785,Piwi | | ArgoN, ArgoL1, PAZ, PIWI | |
| 36. | | TaAGO10a | | Traes_6AS_FBB2AFAAB.1 | | Piwi,ArgoN,PAZ,ArgoL1,ArgoL2,ArgoMid | | DUF1785,PAZ,Piwi | | ArgoN, ArgoL1, PAZ, PIWI | |
| 37. | | TaAGO10b | | Traes_6DS_9DD64BD48.1 | | Piwi,PAZ,ArgoL1,ArgoL2,ArgoMid | | DUF1785,PAZ,Piwi | | ArgoL1, PAZ, PIWI | |
| 38. | | TaAGO10c | | Traes_7AL_96766587F.2 | | Piwi,ArgoL2,ArgoMid,PAZ | | Piwi | | PAZ, PIWI | |
| 39. | | TaAGO10d | | Traes_7DL_C538856D4.1 | | Piwi,PAZ,ArgoL2,ArgoMid | | PAZ,Piwi | | PAZ, PIWI | |
| RDR | | | | | | | | | | | |
| 1. | | TaRDR1a | | Traes_6DL_4B89E8742.2 | | RdRP | | RRM | | RRM_SF,RdRP | |
| 2. | | TaRDR1b | | Traes_6BL_78BEF51DD.1 | | RdRP | | transmembrane region | | RdRP | |
| 3. | | TaRDR1c | | Traes_6AL_393C6B853.1 | | RdRP | | RRM | | RRM_SF,RdRP | |
| 4. | | TaRDR1d | | Traes_6BL_0A9D15EDC.2 | | RdRP | | no domains | | RdRP | |
| 5. | | TaRDR1e | | Traes_6BL_0BB5C493D.1 | | RdRP | | low complexity region | | RdRP | |
| 6. | | TaRDR1f | | Traes_6AL_13BC97E04.1 | | RdRP | | RRM | | RRM_SF,RdRP | |
| 7. | | TaRDR1g | | Traes_6BL_DF680C2AF.1 | | RdRP | | low complexity region | | RdRP | |
| 8. | | TaRDR2a | | Traes_2DL_6DB81005E.1 | | RdRP | | no domains | | RdRP | |
| 9. | | TaRDR2b | | Traes_4AS_8D6311711.1 | | RdRP | | low complexity region | | RdRP | |
| 10. | | TaRDR2c | | Traes_4DL_2E9CE89D9.2 | | RdRP | | low complexity region | | RdRP | |
| 11. | | TaRDR2d | | Traes_4DL_A54C80661.1 | | RdRP | | low complexity region | | RdRP | |
| 12. | | TaRDR3 | | Traes_7BL_8CEC8F99B.2 | | RdRP | | no domains | | RdRP | |
| 13. | | TaRDR4 | | Traes_3AS_F27BB108C.2 | | RdRP | | no domains | | RdRP | |
| 14. | | TaRDR5 | | Traes_3B_2C6DB84FB.2 | | RdRP | | no domains | | RdRP | |
| 15. | | TaRDR6a | | Traes_3B_DC77B5E89.1 | | RdRP | | low complexity region | | RdRP | |
| 16. | | TaRDR6b | | Traes_3DL_F32B49981.1 | | RdRP | | low complexity region | | RdRP | |

**Table S2.** List of the number of alpha-helices, beta-strands at 3-D structure level, and Ka/Ks ratios of the RNAi-dependent pathway proteins in wheat.

| Gene No. | Gene Name | Alpha helix | Beta strand | S | N | Ks | Ka | Ka/Ks |
| --- | --- | --- | --- | --- | --- | --- | --- | --- |
| TaDCLs | | | |  |  |  |  |  |
| 1. | TaDCL1a | 14 | 19 | 1110.2 | 3417.4 | 5.9621 | 0.1956 | 0.0328 |
| 2. | TaDCL1b | 12 | 16 | 1220.1 | 3729.9 | 4.5494 | 0.1594 | 0.035 |
| 3. | TaDCL3a | 13 | 13 | 1146.8 | 3458.2 | 8.0429 | 0.535 | 0.0665 |
| 4. | TaDCL3b | 12 | 17 | 1035.2 | 3194.8 | 5.8129 | 0.5263 | 0.0905 |
| 5. | TaDCL3c | 11 | 16 | 1064.1 | 3396.9 | 6.0653 | 0.5116 | 0.0843 |
| 6. | TaDCL3d | 11 | 26 | 1066.7 | 3394.3 | 6.2513 | 0.5125 | 0.082 |
| 7. | TaDCL4 | 6 | 22 | 991.9 | 3055.1 | 7.4467 | 0.5061 | 0.068 |
| TaAGOs | | | |  |  |  |  |  |
| 1 | TaAGO1a | 14 | 16 | 730.9 | 2401.1 | 3.5511 | 0.1683 | 0.0474 |
| 2. | TaAGO1b | 9 | 12 | 436.2 | 1495.8 | 3.9734 | 0.1019 | 0.0257 |
| 3. | TaAGO1c | 21 | 16 | 723.3 | 2171.7 | 5.6516 | 0.1661 | 0.0294 |
| 4. | TaAGO1d | 14 | 15 | 663.2 | 2057.8 | 5.8631 | 0.1581 | 0.027 |
| 5. | TaAGO1e | 8 | 12 | 516.9 | 1583.1 | 6.9366 | 0.2395 | 0.0345 |
| 6. | TaAGO1f | 12 | 13 | 746.3 | 2385.7 | 4.3352 | 0.2109 | 0.0487 |
| 7. | TaAGO1g | 18 | 14 | 737.3 | 2391.7 | 4.1633 | 0.2074 | 0.0498 |
| 8. | TaAGO1h | 16 | 11 | 744.6 | 2381.4 | 4.1334 | 0.2081 | 0.0503 |
| 9. | TaAGO1i | 18 | 13 | 745.3 | 2392.7 | 4.4973 | 0.1753 | 0.039 |
| 10. | TaAGO1j | 18 | 16 | 605.8 | 1980.2 | 5.7624 | 0.1208 | 0.021 |
| 11. | TaAGO1k | 18 | 14 | 745.4 | 2377.6 | 4.6413 | 0.1751 | 0.0377 |
| 12. | TaAGO2a | 20 | 13 | 667.4 | 2125.6 | 52.6804 | 0.5416 | 0.0103 |
| 13. | TaAGO2b | 19 | 13 | 574.5 | 1897.5 | 54.1759 | 0.534 | 0.0099 |
| 14. | TaAGO3 | 18 | 12 | 584.7 | 1833.3 | 51.7052 | 0.6552 | 0.0127 |
| 15. | TaAGO4a | 15 | 14 | 666.1 | 2081.9 | 10.1484 | 0.2656 | 0.0262 |
| 16. | TaAGO4b | 15 | 12 | 532.2 | 1699.8 | 9.4644 | 0.2917 | 0.0308 |
| 17. | TaAGO4c | 16 | 15 | 651 | 2052 | 8.3366 | 0.272 | 0.0326 |
| 18. | TaAGO5a | 19 | 15 | 576.7 | 1838.3 | 53.4683 | 0.3044 | 0.0057 |
| 19. | TaAGO5b | 26 | 15 | 594.9 | 1874.1 | 19.5146 | 0.3105 | 0.0159 |
| 20. | TaAGO5c | 17 | 16 | 510 | 1644 | 3.8818 | 0.3107 | 0.08 |
| 21. | TaAGO5d | 18 | 15 | 606.6 | 1931.4 | 6.238 | 0.3223 | 0.0517 |
| 22. | TaAGO5e | 18 | 14 | 647.7 | 2013.3 | 5.7949 | 0.3439 | 0.0594 |
| 23. | TaAGO5f | 14 | 16 | 598 | 1862 | 15.7602 | 0.4623 | 0.0293 |
| 24. | TaAGO5g | 18 | 16 | 608.9 | 1875.1 | 17.263 | 0.4763 | 0.0276 |
| 25. | TaAGO6a | 16 | 13 | 517.1 | 1753.9 | 10.0012 | 0.3668 | 0.0367 |
| 26. | TaAGO6b | 21 | 11 | 604.6 | 1996.4 | 6.9925 | 0.3086 | 0.0441 |
| 27. | TaAGO6c | 21 | 15 | 604.6 | 1996.4 | 6.5445 | 0.3099 | 0.0474 |
| 28. | TaAGO6d | 18 | 11 | 606.2 | 1994.8 | 14.8582 | 0.3073 | 0.0207 |
| 29. | TaAGO6e | 18 | 11 | 522.3 | 1670.7 | 3.7717 | 0.417 | 0.1106 |
| 30. | TaAGO7a | 21 | 13 | 683.9 | 2034.1 | 4.4958 | 0.3115 | 0.0693 |
| 31. | TaAGO7b | 22 | 15 | 730.1 | 2155.9 | 4.8792 | 0.3455 | 0.0708 |
| 32. | TaAGO8 | 21 | 13 | 583.9 | 1909.1 | 13.9658 | 0.3251 | 0.0233 |
| 33. | TaAGO9a | 19 | 12 | 655.8 | 2023.2 | 7.1496 | 0.277 | 0.0387 |
| 34. | TaAGO9b | 19 | 13 | 661.5 | 2017.5 | 9.5374 | 0.2762 | 0.029 |
| 35. | TaAGO9c | 20 | 12 | 662 | 2017 | 10.2767 | 0.2774 | 0.027 |
| 36. | TaAGO10a | 20 | 14 | 730.9 | 2092.1 | 46.4767 | 0.1992 | 0.0043 |
| 37. | TaAGO10b | 17 | 14 | 678.2 | 1928.8 | 46.6897 | 0.2261 | 0.0048 |
| 38. | TaAGO10c | 15 | 13 | 420.2 | 1313.8 | 6.2111 | 0.0901 | 0.0145 |
| 39. | TaAGO10d | 18 | 13 | 483.5 | 1508.5 | 5.3154 | 0.0961 | 0.0181 |
| TaRDRs | | | |  |  |  |  |  |
| 1. | TaRDR1a | 11 | 23 | 760.5 | 2536.5 | 51.3665 | 0.3171 | 0.0062 |
| 2. | TaRDR1b | 19 | 21 | 511.2 | 1651.8 | 54.0565 | 0.2924 | 0.0054 |
| 3. | TaRDR1c | 11 | 12 | 754.9 | 2542.1 | 11.6819 | 0.32 | 0.0274 |
| 4. | TaRDR1d | 14 | 16 | 346.2 | 1096.8 | 11.6879 | 0.2154 | 0.0184 |
| 5. | TaRDR1e | 1 | 3 | 227 | 751 | 55.1393 | 0.2655 | 0.0048 |
| 6. | TaRDR1f | 8 | 13 | 765.5 | 2531.5 | 11.5142 | 0.3213 | 0.0279 |
| 7. | TaRDR1g | 13 | 17 | 640.6 | 2164.4 | 12.5809 | 0.3025 | 0.024 |
| 8. | TaRDR2a | 12 | 16 | 531.5 | 1649.5 | 3.3593 | 0.3926 | 0.1169 |
| 9. | TaRDR2b | 12 | 15 | 852.4 | 2495.6 | 5.2929 | 0.4414 | 0.0834 |
| 10. | TaRDR2c | 10 | 10 | 849.9 | 2495.1 | 8.3804 | 0.4475 | 0.0534 |
| 11. | TaRDR2d | 13 | 10 | 657.3 | 1922.7 | 7.1922 | 0.4734 | 0.0658 |
| 12. | TaRDR3 | 12 | 11 | 423.6 | 1394.4 | 4.154 | 0.4651 | 0.112 |
| 13. | TaRDR4 | 6 | 8 | 245.9 | 852.1 | 53.1959 | 0.4549 | 0.0086 |
| 14. | TaRDR5 | 14 | 18 | 356.5 | 1215.5 | 3.0679 | 0.4362 | 0.1422 |
| 15. | TaRDR6a | 18 | 15 | 691.3 | 2038.7 | 46.2402 | 0.2995 | 0.0065 |
| 16. | TaRDR6b | 16 | 14 | 401.8 | 1215.2 | 23.9668 | 0.236 | 0.0098 |

**Table S3.** Percentage of three groups of RNA silencing genes involved in the different cellular locations in wheat (*T.aestivum*)

|  | Names of subcellular locations | | | | | | | | | |
| --- | --- | --- | --- | --- | --- | --- | --- | --- | --- | --- |
|  | **extra** | **cytos** | **membr** | **ER** | **mito** | **golgi** | **plast** | **nucl** | **vacu** | **pero** |
| **DCL** | 0% | 71.4% | 14.3% | 0% | 0% | 0% | 14.3% | 0% | 0% | 0% |
| **AGO** | 2.56% | 87.2% | 7.69% | 0% | 20.5% | 0% | 33.3% | 2.56% | 2.56% | 0% |
| **RDR** | 6.25% | 87.5% | 0% | 0% | 12.5% | 0% | 31.2% | 0% | 6.25% | 0% |

extra(extracellular), cytos (cytpsol),membr (membrane);ER (endoplasmic reticulum); mito(mitochondria);golgi (golgi apparatus);plast (plastid);nucl (nuclear);vacu (vacuole);pero (peroxisome)

**Table S4.** Distribution of TF families those regulating RNAi genes in wheat (*T.aestivum*)

| **Sl No.** | **TF Families** | **Count** | **Percent (%)** |
| --- | --- | --- | --- |
| 1 | ERF | 157 | 41.87 |
| 2 | MIKC-MADS | 38 | 10.13 |
| 3 | C2H2 | 34 | 9.07 |
| 4 | BBR-BPC | 18 | 4.80 |
| 5 | MYB | 17 | 4.53 |
| 6 | Dof | 15 | 4.00 |
| 7 | LBD | 15 | 4.00 |
| 8 | CPP | 10 | 2.67 |
| 9 | AP2 | 10 | 2.67 |
| 10 | Nin-like | 6 | 1.60 |
| 11 | ARR-B | 6 | 1.60 |
| 12 | GATA | 3 | 0.80 |
| 13 | bZIP | 4 | 1.07 |
| 14 | TCP | 4 | 1.07 |
| 15 | NAC | 5 | 1.33 |
| 16 | HD-ZIP | 4 | 1.07 |
| 17 | SBP | 4 | 1.07 |
| 18 | C3H | 4 | 1.07 |
| 19 | G2-like | 3 | 0.80 |
| 20 | bHLH | 4 | 1.07 |
| 21 | EIL | 3 | 0.80 |
| 22 | CAMTA | 2 | 0.53 |
| 23 | LFY | 2 | 0.53 |
| 24 | B3 | 3 | 0.80 |
| 25 | ZF-HD | 2 | 0.53 |
| 26 | WOX | 1 | 0.27 |
| 27 | Trihelix | 1 | 0.27 |
| Total |  | 375 | 100 |
