# Supplementary material for: Comprehensive In Silico Analysis of RNA Silencing-Related Genes and Their Regulatory Elements in Wheat (Triticum aestivum L.)
Source: Biomed Res Int. 2022 Sep 19;2022:4955209. doi: 10.1155/2022/4955209 (PMC9513535; doi:10.1155/2022/4955209)
Supplement: Supplementary 2 — Data S1: protein sequences of the identified DCL genes in wheat. Data S2: protein sequences of the identified AGO genes in wheat. Data S3: protein sequences of the identified RDR genes in wheat. Data S4: list of transcript factors and their families regulating the predicted RNAi-based genes. Data S5: list of cis-regulatory elements associated with the TaDCL protein families. Data S6: list of cis-regulatory elements associated with the TaAGO protein families. Data S7: list of cis-regulatory elements associated with the TaRDR protein families. [file 4955209.f2.zip › Data S4 TF-list375.pdf]

Data S5: List of TF families of the wheat(*T.aestivum*) of RNAi genes

TF_ID	TF	Family	Target(RNAi genes)
TRAES3BF001900070CFD_t1	TRAES3BF001900070CFD_g	bZIP	Traes_2BL_7713B3533
TRAES3BF014900010CFD_t1	TRAES3BF014900010CFD_g	TCP	Traes_7AL_1BAB53DCE
TRAES3BF015800060CFD_t1	TRAES3BF015800060CFD_g	Trihelix	Traes_3DL_F32B49981
TRAES3BF017100030CFD_t1	TRAES3BF017100030CFD_g	ERF	Traes_4DL_2E9CE89D9
TRAES3BF018800030CFD_t1	TRAES3BF018800030CFD_g	MYB	Traes_4AS_8D6311711
TRAES3BF021600020CFD_t1	TRAES3BF021600020CFD_g	MIKC_MADS	Traes_5DL_672EE3605
TRAES3BF064300010CFD_t1	TRAES3BF064300010CFD_g	C2H2	Traes_3AS_F27BB108C
TRAES3BF075600040CFD_t1	TRAES3BF075600040CFD_g	ERF	Traes_1AL_E7144546E
TRAES3BF075600050CFD_t1	TRAES3BF075600050CFD_g	ERF	Traes_2AL_DFE4C65F6
TRAES3BF076800040CFD_t1	TRAES3BF076800040CFD_g	G2-like	Traes_2DL_A77212060
TRAES3BF084400010CFD_t1	TRAES3BF084400010CFD_g	LBD	Traes_4BL_B3A1B8342
TRAES3BF094200030CFD_t1	TRAES3BF094200030CFD_g	ARR-B	Traes_5BL_F505BF164
TRAES3BF094600010CFD_t1	TRAES3BF094600010CFD_g	C2H2	Traes_7AL_D88450A3C
TRAES3BF097400030CFD_t1	TRAES3BF097400030CFD_g	NAC	Traes_4AL_7CC35DF1D
TRAES3BF099600130CFD_t1	TRAES3BF099600130CFD_g	bZIP	Traes_2BL_7713B3533
TRAES3BF107400040CFD_t1	TRAES3BF107400040CFD_g	ARR-B	Traes_5BL_F505BF164
TRAES3BF182900010CFD_t1	TRAES3BF182900010CFD_g	C2H2	Traes_6AS_FBB2AFAAB
Traes_1AL_1B5F51626.1	Traes_1AL_1B5F51626	MIKC_MADS	Traes_6BL_9CFA54D4A
Traes_1AL_6B108514B.1	Traes_1AL_6B108514B	MIKC_MADS	Traes_7BL_8CEC8F99B
Traes_1AL_A9FB6BF52.1	Traes_1AL_A9FB6BF52	ERF	Traes_7AL_1BAB53DCE
Traes_1AS_1EE692FDC.1	Traes_1AS_1EE692FDC	MYB	Traes_5BL_F611D65E0
Traes_1AS_61D017632.2	Traes_1AS_61D017632	MYB	Traes_6DL_804FB7F75
Traes_1BS_403DBC53C.1	Traes_1BS_403DBC53C	MYB	Traes_6DL_804FB7F75
Traes_1BS_92EA1F290.1	Traes_1BS_92EA1F290	MYB	Traes_5BL_F611D65E0
Traes_1DS_5BAD8947E.1	Traes_1DS_5BAD8947E	MYB	Traes_5BL_F611D65E0
Traes_1DS_BB8508CC6.1	Traes_1DS_BB8508CC6	LBD	Traes_4BL_B3A1B8342
Traes_2AL_E5A9615E2.3	Traes_2AL_E5A9615E2	ERF	Traes_7AL_1BAB53DCE
Traes_2AL_F24D031AA.1	Traes_2AL_F24D031AA	ERF	Traes_6DS_9DD64BD48
Traes_2AL_FC6DD1383.1	Traes_2AL_FC6DD1383	ERF	Traes_6DL_58620B158
Traes_2AS_40FA27AE7.1	Traes_2AS_40FA27AE7	MYB	Traes_5BL_F611D65E0
Traes_2AS_4C4190DEB.2	Traes_2AS_4C4190DEB	CAMTA	Traes_2BL_7713B3533
Traes_2BL_1E57B73B21.5	Traes_2BL_1E57B73B21	ERF	Traes_1AL_E7144546E
Traes_2BL_569371098.2	Traes_2BL_569371098	AP2	Traes_1DL_64B330BBB
Traes_2BL_8DEC0EFBF.2	Traes_2BL_8DEC0EFBF	LFY	Traes_6BL_0BB5C493D
Traes_2BL_9CD6E043A.2	Traes_2BL_9CD6E043A	ERF	Traes_2AL_3F3117458
Traes_2BL_B397F2CE3.1	Traes_2BL_B397F2CE3	ERF	Traes_4BL_B3A1B8342
Traes_2BL_EBA73F94F.1	Traes_2BL_EBA73F94F	AP2	Traes_1AL_E7144546E
Traes_2BS_4818EA1FF.1	Traes_2BS_4818EA1FF	MIKC_MADS	Traes_4AS_8D6311711
Traes_2BS_B1A73C7A8.1	Traes_2BS_B1A73C7A8	ERF	Traes_4DL_2E9CE89D9
Traes_2BS_DD07C2EE6.2	Traes_2BS_DD07C2EE6	C2H2	Traes_6AL_317133B3F
Traes_2BS_F1B450FA4.2	Traes_2BS_F1B450FA4	MYB	Traes_4AS_8D6311711
Traes_2DL_D39684C41.1	Traes_2DL_D39684C41	MYB	Traes_6AL_13BC97E04
Traes_3AL_00FAACD5D.1	Traes_3AL_00FAACD5D	C2H2	Traes_3DS_57EA31670
Traes_3AS_09025DA2E.1	Traes_3AS_09025DA2E	MYB	Traes_6DL_804FB7F75
Traes_3AS_55E9080C2.2	Traes_3AS_55E9080C2	MIKC_MADS	Traes_5DL_672EE3605

Traes_3AS_88AAED4BB.1	Traes_3AS_88AAED4BB	C2H2	Traes_2AL_3F3117458
Traes_3DL_2C29058E6.1	Traes_3DL_2C29058E6	B3	Traes_2DL_A77212060
Traes_3DL_C686CE2E3.2	Traes_3DL_C686CE2E3	NAC	Traes_4AL_7CC35DF1D
Traes_4AL_0BB0A30D1.2	Traes_4AL_0BB0A30D1	bHLH	Traes_2BL_7713B3533
Traes_4AL_5062917C4.1	Traes_4AL_5062917C4	SBP	Traes_6BL_78BEF51DD
Traes_4AL_72ECC7747.1	Traes_4AL_72ECC7747	G2-like	Traes_5AL_07EFD5712
Traes_4AL_A02408BC8.2	Traes_4AL_A02408BC8	BBR-BPC	Traes_7AL_D88450A3C
Traes_4AS_0D75149B5.1	Traes_4AS_0D75149B5	ERF	Traes_6DS_9DD64BD48
Traes_4AS_48979CBE1.2	Traes_4AS_48979CBE1	BBR-BPC	Traes_2AL_2512A7F91
Traes_4AS_501676A91.1	Traes_4AS_501676A91	C2H2	Traes_2AL_3F3117458
Traes_4BL_085791CB2.1	Traes_4BL_085791CB2	WOX	Traes_3DL_F32B49981
Traes_4BL_78DD63002.1	Traes_4BL_78DD63002	HD-ZIP	Traes_6DL_58620B158
Traes_4BL_7911992DE.1	Traes_4BL_7911992DE	Dof	Traes_1AL_E7144546E
Traes_4BL_941E7DE4D.2	Traes_4BL_941E7DE4D	ARR-B	Traes_5BL_F505BF164
Traes_4BL_A37F6FD08.3	Traes_4BL_A37F6FD08	CAMTA	Traes_7AL_96766587F
Traes_4BS_929E7B27B.2	Traes_4BS_929E7B27B	CPP	Traes_5AL_07EFD5712
Traes_4DL_AE5093871.1	Traes_4DL_AE5093871	BBR-BPC	Traes_1AL_E7144546E
Traes_4DL_C083C804E.1	Traes_4DL_C083C804E	EIL	Traes_7AL_D88450A3C
Traes_4DS_2E95B3A30.2	Traes_4DS_2E95B3A30	CPP	Traes_7AL_1BAB53DCE
Traes_4DS_F3750ABFD.1	Traes_4DS_F3750ABFD	ZF-HD	Traes_6AL_317133B3F
Traes_5AL_0D4BDDDCD.1	Traes_5AL_0D4BDDDCD	bHLH	Traes_2BL_7713B3533
Traes_5AL_315285EFC.1	Traes_5AL_315285EFC	Nin-like	Traes_2AL_3F3117458
Traes_5AL_315285EFC1.1	Traes_5AL_315285EFC1	Nin-like	Traes_5AL_72A7552B9
Traes_5AL_B65EF5518.3	Traes_5AL_B65EF5518	EIL	Traes_7AL_D88450A3C
Traes_5AS_3D6AA43B5.1	Traes_5AS_3D6AA43B5	B3	Traes_3AL_562D6614F
Traes_5BL_096B0BDA8.2	Traes_5BL_096B0BDA8	C2H2	Traes_2AL_3F3117458
Traes_5BL_632EBAD09.1	Traes_5BL_632EBAD09	MYB	Traes_2BL_93099ACF4
Traes_5BL_7F56360BF.1	Traes_5BL_7F56360BF	C2H2	Traes_3AS_F27BB108C
Traes_5BL_9627436AE.1	Traes_5BL_9627436AE	MIKC_MADS	Traes_4AS_8D6311711
Traes_5BL_9C32B27E2.1	Traes_5BL_9C32B27E2	HD-ZIP	Traes_7AL_96766587F
Traes_5BL_A5532B750.1	Traes_5BL_A5532B750	bZIP	Traes_2BL_7713B3533
Traes_5BL_AAC9C7238.2	Traes_5BL_AAC9C7238	bHLH	Traes_2BL_7713B3533
Traes_5BL_B6051DD37.2	Traes_5BL_B6051DD37	EIL	Traes_2AL_2512A7F91
Traes_5BL_F258582BB.1	Traes_5BL_F258582BB	GATA	Traes_2DS_4CC8FD7E3
Traes_5BL_F5D379AFC.1	Traes_5BL_F5D379AFC	ERF	Traes_4AS_8D6311711
Traes_5BL_FBC7AF288.1	Traes_5BL_FBC7AF288	ERF	Traes_6DS_9DD64BD48
Traes_5BS_A5532B750.1	Traes_5BS_A5532B750	bZIP	Traes_2BL_7713B3533
Traes_5DL_1868E2A6C.1	Traes_5DL_1868E2A6C	MYB	Traes_6DL_4B89E8742
Traes_5DL_92CBBE968.1	Traes_5DL_92CBBE968	MYB	Traes_2AL_2512A7F91
Traes_5DL_96F9EED93.2	Traes_5DL_96F9EED93	HD-ZIP	Traes_7AL_96766587F
Traes_5DL_9CC4EC839.1	Traes_5DL_9CC4EC839	MIKC_MADS	Traes_4AS_8D6311711
Traes_5DS_AACA2EBA3.2	Traes_5DS_AACA2EBA3	MYB	Traes_6AL_13BC97E04
Traes_6AL_1F7DAC5FA.2	Traes_6AL_1F7DAC5FA	MIKC_MADS	Traes_6BL_0BB5C493D
Traes_6AL_8BA1FF8B2.1	Traes_6AL_8BA1FF8B2	NAC	Traes_6DL_58620B158
Traes_6AL_A93C6F2FC.1	Traes_6AL_A93C6F2FC	MIKC_MADS	Traes_6BL_9CFA54D4A
Traes_6AS_70AD4B0D5.1	Traes_6AS_70AD4B0D5	C3H	Traes_6BL_9CFA54D4A
Traes_6BL_7C6B17284.2	Traes_6BL_7C6B17284	MIKC_MADS	Traes_6BL_0BB5C493D

Traes_6DL_136DE13FB.1	Traes_6DL_136DE13FB	TCP	Traes_5DL_672EE3605
Traes_6DL_3019F176A.1	Traes_6DL_3019F176A	ERF	Traes_2AL_3F3117458
Traes_6DL_88DFCF552.1	Traes_6DL_88DFCF552	C2H2	Traes_1BL_7C037D478
Traes_6DL_8D95F127B.1	Traes_6DL_8D95F127B	HD-ZIP	Traes_6DL_4B89E8742
Traes_6DL_991931CF9.2	Traes_6DL_991931CF9	MYB	Traes_6DL_4B89E8742
Traes_6DL_D1C1DBD34.1	Traes_6DL_D1C1DBD34	MIKC_MADS	Traes_6BL_0BB5C493D
Traes_6DL_DD82BD107.2	Traes_6DL_DD82BD107	ERF	Traes_7AL_1BAB53DCE
Traes_6DL_EF6D5D4FA.1	Traes_6DL_EF6D5D4FA	Dof	Traes_6BL_9CFA54D4A
Traes_6DL_FA861D610.2	Traes_6DL_FA861D610	ERF	Traes_6AS_FBB2AFAAB
Traes_6DL_FC5CFB787.3	Traes_6DL_FC5CFB787	TCP	Traes_2AL_3F3117458
Traes_6DS_2B2F9C290.2	Traes_6DS_2B2F9C290	C2H2	Traes_7AL_1BAB53DCE
Traes_7AL_D45376F32.2	Traes_7AL_D45376F32	G2-like	Traes_5BL_F611D65E0
Traes_7AL_EA6F4FFDE.2	Traes_7AL_EA6F4FFDE	GATA	Traes_4DL_A54C80661
Traes_7AS_0F019FB93.2	Traes_7AS_0F019FB93	SBP	Traes_6BL_78BEF51DD
Traes_7DL_DDCC09B24.1	Traes_7DL_DDCC09B24	MIKC_MADS	Traes_7AL_1BAB53DCE
Traes_7DS_433704E8E.1	Traes_7DS_433704E8E	ERF	Traes_4DL_2E9CE89D9
Traes_7DS_433704E8E1.1	Traes_7DS_433704E8E1	ERF	Traes_4DL_2E9CE89D9
Traes_7DS_FE8BC1125.1	Traes_7DS_FE8BC1125	BBR-BPC	Traes_7BL_8CEC8F99B
TRAES3BF017100030CFD_t1	TRAES3BF017100030CFD_g	ERF	Traes_4DS_88D2821C6
TRAES3BF017100030CFD_t1	TRAES3BF017100030CFD_g	ERF	Traes_5BL_F611D65E0
TRAES3BF017100030CFD_t1	TRAES3BF017100030CFD_g	ERF	Traes_1BL_7C037D478
TRAES3BF017100030CFD_t1	TRAES3BF017100030CFD_g	ERF	Traes_1AL_E7144546E
TRAES3BF017100030CFD_t1	TRAES3BF017100030CFD_g	ERF	Traes_6AL_13BC97E04
TRAES3BF017100030CFD_t1	TRAES3BF017100030CFD_g	ERF	Traes_6DS_9DD64BD48
TRAES3BF017100030CFD_t1	TRAES3BF017100030CFD_g	ERF	Traes_4BL_B3A1B8342
TRAES3BF017100030CFD_t1	TRAES3BF017100030CFD_g	ERF	Traes_2AL_3F3117458
TRAES3BF017100030CFD_t1	TRAES3BF017100030CFD_g	ERF	Traes_2AL_DFE4C65F6
TRAES3BF017100030CFD_t1	TRAES3BF017100030CFD_g	ERF	Traes_7AL_1BAB53DCE
TRAES3BF017100030CFD_t1	TRAES3BF017100030CFD_g	ERF	Traes_4AS_8D6311711
TRAES3BF017100030CFD_t1	TRAES3BF017100030CFD_g	ERF	Traes_6AL_317133B3F
TRAES3BF021600020CFD_t1	TRAES3BF021600020CFD_g	MIKC_MADS	Traes_7BL_8CEC8F99B
TRAES3BF021600020CFD_t1	TRAES3BF021600020CFD_g	MIKC_MADS	Traes_6DL_4B89E8742
TRAES3BF021600020CFD_t1	TRAES3BF021600020CFD_g	MIKC_MADS	Traes_6BL_9CFA54D4A
TRAES3BF021600020CFD_t1	TRAES3BF021600020CFD_g	MIKC_MADS	Traes_5BL_F611D65E0
TRAES3BF021600020CFD_t1	TRAES3BF021600020CFD_g	MIKC_MADS	Traes_7DL_C538856D4
TRAES3BF064300010CFD_t1	TRAES3BF064300010CFD_g	C2H2	Traes_2AL_3F3117458
TRAES3BF075600040CFD_t1	TRAES3BF075600040CFD_g	ERF	Traes_6DS_9DD64BD48
TRAES3BF075600040CFD_t1	TRAES3BF075600040CFD_g	ERF	Traes_1BL_7C037D478
TRAES3BF075600040CFD_t1	TRAES3BF075600040CFD_g	ERF	Traes_4BL_B3A1B8342
TRAES3BF075600040CFD_t1	TRAES3BF075600040CFD_g	ERF	Traes_2AL_3F3117458
TRAES3BF075600040CFD_t1	TRAES3BF075600040CFD_g	ERF	Traes_4AS_8D6311711
TRAES3BF075600050CFD_t1	TRAES3BF075600050CFD_g	ERF	Traes_2AL_3F3117458
TRAES3BF075600050CFD_t1	TRAES3BF075600050CFD_g	ERF	Traes_1BL_7C037D478
TRAES3BF075600050CFD_t1	TRAES3BF075600050CFD_g	ERF	Traes_1AL_E7144546E
TRAES3BF075600050CFD_t1	TRAES3BF075600050CFD_g	ERF	Traes_4AS_8D6311711
TRAES3BF075600050CFD_t1	TRAES3BF075600050CFD_g	ERF	Traes_4DL_2E9CE89D9
TRAES3BF075600050CFD_t1	TRAES3BF075600050CFD_g	ERF	Traes_4BL_B3A1B8342

TRAES3BF084400010CFD_t1	TRAES3BF084400010CFD_g	LBD	Traes_1AL_E7144546E
TRAES3BF084400010CFD_t1	TRAES3BF084400010CFD_g	LBD	Traes_6DS_9DD64BD48
TRAES3BF084400010CFD_t1	TRAES3BF084400010CFD_g	LBD	Traes_4DL_2E9CE89D9
TRAES3BF084400010CFD_t1	TRAES3BF084400010CFD_g	LBD	Traes_2AL_DFE4C65F6
TRAES3BF084400010CFD_t1	TRAES3BF084400010CFD_g	LBD	Traes_4AS_8D6311711
TRAES3BF084400010CFD_t1	TRAES3BF084400010CFD_g	LBD	Traes_7AL_1BAB53DCE
TRAES3BF084400010CFD_t1	TRAES3BF084400010CFD_g	LBD	Traes_6DL_4B89E8742
TRAES3BF084400010CFD_t1	TRAES3BF084400010CFD_g	LBD	Traes_1BL_7C037D478
TRAES3BF084400010CFD_t1	TRAES3BF084400010CFD_g	LBD	Traes_2BL_7713B3533
TRAES3BF094200030CFD_t1	TRAES3BF094200030CFD_g	ARR-B	Traes_2DL_A77212060
TRAES3BF107400040CFD_t1	TRAES3BF107400040CFD_g	ARR-B	Traes_2DL_A77212060
TRAES3BF182900010CFD_t1	TRAES3BF182900010CFD_g	C2H2	Traes_2AL_3F3117458
TRAES3BF182900010CFD_t1	TRAES3BF182900010CFD_g	C2H2	Traes_3AS_F27BB108C
Traes_1AL_1B5F51626.1	Traes_1AL_1B5F51626	MIKC_MADS	Traes_5DL_672EE3605
Traes_1AL_1B5F51626.1	Traes_1AL_1B5F51626	MIKC_MADS	Traes_7DL_C538856D4
Traes_1AL_1B5F51626.1	Traes_1AL_1B5F51626	MIKC_MADS	Traes_5BL_F611D65E0
Traes_1AL_1B5F51626.1	Traes_1AL_1B5F51626	MIKC_MADS	Traes_7BL_8CEC8F99B
Traes_1AL_1B5F51626.1	Traes_1AL_1B5F51626	MIKC_MADS	Traes_6DL_4B89E8742
Traes_1AL_6B108514B.1	Traes_1AL_6B108514B	MIKC_MADS	Traes_2BL_93099ACF4
Traes_1AL_6B108514B.1	Traes_1AL_6B108514B	MIKC_MADS	Traes_4DL_2E9CE89D9
Traes_1AL_6B108514B.1	Traes_1AL_6B108514B	MIKC_MADS	Traes_1AL_E7144546E
Traes_1AL_6B108514B.1	Traes_1AL_6B108514B	MIKC_MADS	Traes_6BL_9CFA54D4A
Traes_1AL_6B108514B.1	Traes_1AL_6B108514B	MIKC_MADS	Traes_5BL_F611D65E0
Traes_1AL_6B108514B.1	Traes_1AL_6B108514B	MIKC_MADS	Traes_7AL_96766587F
Traes_1AL_6B108514B.1	Traes_1AL_6B108514B	MIKC_MADS	Traes_7DS_4D01B6175
Traes_1AL_6B108514B.1	Traes_1AL_6B108514B	MIKC_MADS	Traes_1AL_095416BC0
Traes_1AL_6B108514B.1	Traes_1AL_6B108514B	MIKC_MADS	Traes_4DL_A54C80661
Traes_1AL_6B108514B.1	Traes_1AL_6B108514B	MIKC_MADS	Traes_7AL_D88450A3C
Traes_1AL_A9FB6BF52.1	Traes_1AL_A9FB6BF52	ERF	Traes_6DL_4B89E8742
Traes_1AL_A9FB6BF52.1	Traes_1AL_A9FB6BF52	ERF	Traes_2AL_DFE4C65F6
Traes_1AL_A9FB6BF52.1	Traes_1AL_A9FB6BF52	ERF	Traes_4AS_8D6311711
Traes_1AL_A9FB6BF52.1	Traes_1AL_A9FB6BF52	ERF	Traes_5BL_F611D65E0
Traes_1AL_A9FB6BF52.1	Traes_1AL_A9FB6BF52	ERF	Traes_6AL_13BC97E04
Traes_1AL_A9FB6BF52.1	Traes_1AL_A9FB6BF52	ERF	Traes_6DS_9DD64BD48
Traes_1AL_A9FB6BF52.1	Traes_1AL_A9FB6BF52	ERF	Traes_2AL_3F3117458
Traes_1AL_A9FB6BF52.1	Traes_1AL_A9FB6BF52	ERF	Traes_6AS_FBB2AFAAB
Traes_1AL_A9FB6BF52.1	Traes_1AL_A9FB6BF52	ERF	Traes_1BL_7C037D478
Traes_1AL_A9FB6BF52.1	Traes_1AL_A9FB6BF52	ERF	Traes_4DL_2E9CE89D9
Traes_1AL_A9FB6BF52.1	Traes_1AL_A9FB6BF52	ERF	Traes_1AL_E7144546E
Traes_1AL_A9FB6BF52.1	Traes_1AL_A9FB6BF52	ERF	Traes_4BL_B3A1B8342
Traes_1DS_BB8508CC6.1	Traes_1DS_BB8508CC6	LBD	Traes_2AL_DFE4C65F6
Traes_1DS_BB8508CC6.1	Traes_1DS_BB8508CC6	LBD	Traes_2BL_7713B3533
Traes_1DS_BB8508CC6.1	Traes_1DS_BB8508CC6	LBD	Traes_1AL_E7144546E
Traes_1DS_BB8508CC6.1	Traes_1DS_BB8508CC6	LBD	Traes_4AS_8D6311711
Traes_2AL_E5A9615E2.3	Traes_2AL_E5A9615E2	ERF	Traes_2BL_7713B3533
Traes_2AL_E5A9615E2.3	Traes_2AL_E5A9615E2	ERF	Traes_2AL_3F3117458
Traes_2AL_E5A9615E2.3	Traes_2AL_E5A9615E2	ERF	Traes_6DL_58620B158

Traes_2AL_E5A9615E2.3	Traes_2AL_E5A9615E2	ERF	Traes_4BL_B3A1B8342
Traes_2AL_E5A9615E2.3	Traes_2AL_E5A9615E2	ERF	Traes_6DS_9DD64BD48
Traes_2AL_E5A9615E2.3	Traes_2AL_E5A9615E2	ERF	Traes_1BL_7C037D478
Traes_2AL_E5A9615E2.3	Traes_2AL_E5A9615E2	ERF	Traes_4AS_8D6311711
Traes_2AL_E5A9615E2.3	Traes_2AL_E5A9615E2	ERF	Traes_2AL_DFE4C65F6
Traes_2AL_E5A9615E2.3	Traes_2AL_E5A9615E2	ERF	Traes_5BL_F611D65E0
Traes_2AL_E5A9615E2.3	Traes_2AL_E5A9615E2	ERF	Traes_4DL_2E9CE89D9
Traes_2AL_E5A9615E2.3	Traes_2AL_E5A9615E2	ERF	Traes_1AL_E7144546E
Traes_2AL_F24D031AA.1	Traes_2AL_F24D031AA	ERF	Traes_4BL_B3A1B8342
Traes_2AL_F24D031AA.1	Traes_2AL_F24D031AA	ERF	Traes_2AL_DFE4C65F6
Traes_2AL_F24D031AA.1	Traes_2AL_F24D031AA	ERF	Traes_4DL_2E9CE89D9
Traes_2AL_F24D031AA.1	Traes_2AL_F24D031AA	ERF	Traes_1BL_7C037D478
Traes_2AL_F24D031AA.1	Traes_2AL_F24D031AA	ERF	Traes_7AL_1BAB53DCE
Traes_2AL_F24D031AA.1	Traes_2AL_F24D031AA	ERF	Traes_3DS_57EA31670
Traes_2AL_F24D031AA.1	Traes_2AL_F24D031AA	ERF	Traes_1AL_E7144546E
Traes_2AL_F24D031AA.1	Traes_2AL_F24D031AA	ERF	Traes_2AL_3F3117458
Traes_2AL_F24D031AA.1	Traes_2AL_F24D031AA	ERF	Traes_4AS_8D6311711
Traes_2AL_F24D031AA.1	Traes_2AL_F24D031AA	ERF	Traes_2BL_7713B3533
Traes_2AL_FC6DD1383.1	Traes_2AL_FC6DD1383	ERF	Traes_7AL_1BAB53DCE
Traes_2AL_FC6DD1383.1	Traes_2AL_FC6DD1383	ERF	Traes_1AL_E7144546E
Traes_2AL_FC6DD1383.1	Traes_2AL_FC6DD1383	ERF	Traes_4AS_8D6311711
Traes_2AL_FC6DD1383.1	Traes_2AL_FC6DD1383	ERF	Traes_1BL_7C037D478
Traes_2AL_FC6DD1383.1	Traes_2AL_FC6DD1383	ERF	Traes_2AL_DFE4C65F6
Traes_2AL_FC6DD1383.1	Traes_2AL_FC6DD1383	ERF	Traes_4BL_B3A1B8342
Traes_2AL_FC6DD1383.1	Traes_2AL_FC6DD1383	ERF	Traes_5BL_F611D65E0
Traes_2AL_FC6DD1383.1	Traes_2AL_FC6DD1383	ERF	Traes_2BL_7713B3533
Traes_2AL_FC6DD1383.1	Traes_2AL_FC6DD1383	ERF	Traes_2AL_3F3117458
Traes_2AL_FC6DD1383.1	Traes_2AL_FC6DD1383	ERF	Traes_4DL_2E9CE89D9
Traes_2AL_FC6DD1383.1	Traes_2AL_FC6DD1383	ERF	Traes_6DS_9DD64BD48
Traes_2BL_1E57B73B21.5	Traes_2BL_1E57B73B21	ERF	Traes_6AS_FBB2AFAAB
Traes_2BL_9CD6E043A.2	Traes_2BL_9CD6E043A	ERF	Traes_4DL_2E9CE89D9
Traes_2BL_9CD6E043A.2	Traes_2BL_9CD6E043A	ERF	Traes_7AL_1BAB53DCE
Traes_2BL_9CD6E043A.2	Traes_2BL_9CD6E043A	ERF	Traes_6DS_9DD64BD48
Traes_2BL_9CD6E043A.2	Traes_2BL_9CD6E043A	ERF	Traes_4BL_B3A1B8342
Traes_2BL_9CD6E043A.2	Traes_2BL_9CD6E043A	ERF	Traes_2BL_7713B3533
Traes_2BL_9CD6E043A.2	Traes_2BL_9CD6E043A	ERF	Traes_1BL_7C037D478
Traes_2BL_9CD6E043A.2	Traes_2BL_9CD6E043A	ERF	Traes_2AL_DFE4C65F6
Traes_2BL_9CD6E043A.2	Traes_2BL_9CD6E043A	ERF	Traes_4AS_8D6311711
Traes_2BL_9CD6E043A.2	Traes_2BL_9CD6E043A	ERF	Traes_1AL_E7144546E
Traes_2BL_B397F2CE3.1	Traes_2BL_B397F2CE3	ERF	Traes_2AL_3F3117458
Traes_2BL_B397F2CE3.1	Traes_2BL_B397F2CE3	ERF	Traes_1BL_7C037D478
Traes_2BL_B397F2CE3.1	Traes_2BL_B397F2CE3	ERF	Traes_1AL_E7144546E
Traes_2BL_B397F2CE3.1	Traes_2BL_B397F2CE3	ERF	Traes_7AL_1BAB53DCE
Traes_2BL_B397F2CE3.1	Traes_2BL_B397F2CE3	ERF	Traes_4AS_8D6311711
Traes_2BL_B397F2CE3.1	Traes_2BL_B397F2CE3	ERF	Traes_6DS_9DD64BD48
Traes_2BL_EBA73F94F.1	Traes_2BL_EBA73F94F	AP2	Traes_6AL_13BC97E04
Traes_2BL_EBA73F94F.1	Traes_2BL_EBA73F94F	AP2	Traes_7AL_D88450A3C

Traes_2BL_EBA73F94F.1	Traes_2BL_EBA73F94F	AP2	Traes_4DL_A54C80661
Traes_2BL_EBA73F94F.1	Traes_2BL_EBA73F94F	AP2	Traes_7AL_96766587F
Traes_2BL_EBA73F94F.1	Traes_2BL_EBA73F94F	AP2	Traes_6BL_9CFA54D4A
Traes_2BL_EBA73F94F.1	Traes_2BL_EBA73F94F	AP2	Traes_5BL_F611D65E0
Traes_2BL_EBA73F94F.1	Traes_2BL_EBA73F94F	AP2	Traes_5AL_07EFD5712
Traes_2BL_EBA73F94F.1	Traes_2BL_EBA73F94F	AP2	Traes_7BL_8CEC8F99B
Traes_2BS_B1A73C7A8.1	Traes_2BS_B1A73C7A8	ERF	Traes_4AS_8D6311711
Traes_2BS_B1A73C7A8.1	Traes_2BS_B1A73C7A8	ERF	Traes_1BL_7C037D478
Traes_2BS_B1A73C7A8.1	Traes_2BS_B1A73C7A8	ERF	Traes_1AL_E7144546E
Traes_2BS_B1A73C7A8.1	Traes_2BS_B1A73C7A8	ERF	Traes_2AL_3F3117458
Traes_2BS_B1A73C7A8.1	Traes_2BS_B1A73C7A8	ERF	Traes_2BL_7713B3533
Traes_2BS_B1A73C7A8.1	Traes_2BS_B1A73C7A8	ERF	Traes_4BL_B3A1B8342
Traes_2BS_B1A73C7A8.1	Traes_2BS_B1A73C7A8	ERF	Traes_6DS_9DD64BD48
Traes_2BS_DD07C2EE6.2	Traes_2BS_DD07C2EE6	C2H2	Traes_1AL_E7144546E
Traes_3AS_55E9080C2.2	Traes_3AS_55E9080C2	MIKC_MADS	Traes_7AS_56569A5AC
Traes_3AS_55E9080C2.2	Traes_3AS_55E9080C2	MIKC_MADS	Traes_6BL_0BB5C493D
Traes_3AS_55E9080C2.2	Traes_3AS_55E9080C2	MIKC_MADS	Traes_2DL_E96DCDCB4
Traes_3AS_55E9080C2.2	Traes_3AS_55E9080C2	MIKC_MADS	Traes_6BL_9CFA54D4A
Traes_3AS_88AAED4BB.1	Traes_3AS_88AAED4BB	C2H2	Traes_6AS_FBB2AFAAB
Traes_3AS_88AAED4BB.1	Traes_3AS_88AAED4BB	C2H2	Traes_3AS_F27BB108C
Traes_4AL_5062917C4.1	Traes_4AL_5062917C4	SBP	Traes_6BL_0A9D15EDC
Traes_4AL_A02408BC8.2	Traes_4AL_A02408BC8	BBR-BPC	Traes_7BL_8CEC8F99B
Traes_4AS_0D75149B5.1	Traes_4AS_0D75149B5	ERF	Traes_2AL_3F3117458
Traes_4AS_0D75149B5.1	Traes_4AS_0D75149B5	ERF	Traes_1AL_E7144546E
Traes_4AS_0D75149B5.1	Traes_4AS_0D75149B5	ERF	Traes_4BL_B3A1B8342
Traes_4AS_0D75149B5.1	Traes_4AS_0D75149B5	ERF	Traes_2AL_DFE4C65F6
Traes_4AS_0D75149B5.1	Traes_4AS_0D75149B5	ERF	Traes_5BL_F611D65E0
Traes_4AS_0D75149B5.1	Traes_4AS_0D75149B5	ERF	Traes_1BL_7C037D478
Traes_4AS_0D75149B5.1	Traes_4AS_0D75149B5	ERF	Traes_7AL_1BAB53DCE
Traes_4AS_0D75149B5.1	Traes_4AS_0D75149B5	ERF	Traes_4AS_8D6311711
Traes_4AS_48979CBE1.2	Traes_4AS_48979CBE1	BBR-BPC	Traes_4DL_A54C80661
Traes_4AS_48979CBE1.2	Traes_4AS_48979CBE1	BBR-BPC	Traes_3DS_57EA31670
Traes_4AS_48979CBE1.2	Traes_4AS_48979CBE1	BBR-BPC	Traes_3AS_3F8424E4E
Traes_4AS_48979CBE1.2	Traes_4AS_48979CBE1	BBR-BPC	Traes_1DL_64B330BBB
Traes_4AS_48979CBE1.2	Traes_4AS_48979CBE1	BBR-BPC	Traes_1AL_E7144546E
Traes_4AS_48979CBE1.2	Traes_4AS_48979CBE1	BBR-BPC	Traes_7BL_8CEC8F99B
Traes_4AS_501676A91.1	Traes_4AS_501676A91	C2H2	Traes_3AS_F27BB108C
Traes_4AS_501676A91.1	Traes_4AS_501676A91	C2H2	Traes_7AL_96766587F
Traes_4BL_7911992DE.1	Traes_4BL_7911992DE	Dof	Traes_6AL_13BC97E04
Traes_4BL_7911992DE.1	Traes_4BL_7911992DE	Dof	Traes_5AL_07EFD5712
Traes_4BL_7911992DE.1	Traes_4BL_7911992DE	Dof	Traes_6DL_4B89E8742
Traes_4BL_7911992DE.1	Traes_4BL_7911992DE	Dof	Traes_7DS_4D01B6175
Traes_4BL_7911992DE.1	Traes_4BL_7911992DE	Dof	Traes_3DL_2DC78B18A
Traes_4BL_7911992DE.1	Traes_4BL_7911992DE	Dof	Traes_4DL_A54C80661
Traes_4BL_7911992DE.1	Traes_4BL_7911992DE	Dof	Traes_2AL_2512A7F91
Traes_4BL_7911992DE.1	Traes_4BL_7911992DE	Dof	Traes_6BL_9CFA54D4A
Traes_4BL_7911992DE.1	Traes_4BL_7911992DE	Dof	Traes_7AL_96766587F

Traes_4BS_929E7B27B.2	Traes_4BS_929E7B27B	CPP	Traes_5BL_F505BF164
Traes_4BS_929E7B27B.2	Traes_4BS_929E7B27B	CPP	Traes_7AL_1BAB53DCE
Traes_4BS_929E7B27B.2	Traes_4BS_929E7B27B	CPP	Traes_4DL_2E9CE89D9
Traes_4BS_929E7B27B.2	Traes_4BS_929E7B27B	CPP	Traes_3DS_57EA31670
Traes_4DL_AE5093871.1	Traes_4DL_AE5093871	BBR-BPC	Traes_4DL_A54C80661
Traes_4DL_AE5093871.1	Traes_4DL_AE5093871	BBR-BPC	Traes_7BL_8CEC8F99B
Traes_4DL_AE5093871.1	Traes_4DL_AE5093871	BBR-BPC	Traes_2AL_2512A7F91
Traes_4DL_AE5093871.1	Traes_4DL_AE5093871	BBR-BPC	Traes_3DS_57EA31670
Traes_4DL_AE5093871.1	Traes_4DL_AE5093871	BBR-BPC	Traes_3AS_3F8424E4E
Traes_4DL_AE5093871.1	Traes_4DL_AE5093871	BBR-BPC	Traes_1DL_64B330BBB
Traes_4DL_C083C804E.1	Traes_4DL_C083C804E	EIL	Traes_2BL_24111235C
Traes_4DS_2E95B3A30.2	Traes_4DS_2E95B3A30	CPP	Traes_5AL_07EFD5712
Traes_4DS_2E95B3A30.2	Traes_4DS_2E95B3A30	CPP	Traes_5BL_F505BF164
Traes_4DS_2E95B3A30.2	Traes_4DS_2E95B3A30	CPP	Traes_3DS_57EA31670
Traes_4DS_2E95B3A30.2	Traes_4DS_2E95B3A30	CPP	Traes_4DL_2E9CE89D9
Traes_5AL_315285EFC.1	Traes_5AL_315285EFC	Nin-like	Traes_5AL_72A7552B9
Traes_5AL_315285EFC.1	Traes_5AL_315285EFC	Nin-like	Traes_2BL_24111235C
Traes_5AL_315285EFC.1	Traes_5AL_315285EFC	Nin-like	Traes_4BL_B3A1B8342
Traes_5AL_315285EFC1.1	Traes_5AL_315285EFC1	Nin-like	Traes_4BL_B3A1B8342
Traes_5AL_315285EFC1.1	Traes_5AL_315285EFC1	Nin-like	Traes_2BL_24111235C
Traes_5AL_315285EFC1.1	Traes_5AL_315285EFC1	Nin-like	Traes_2AL_3F3117458
Traes_5AS_3D6AA43B5.1	Traes_5AS_3D6AA43B5	B3	Traes_3AS_8EE711E2C
Traes_5BL_096B0BDA8.2	Traes_5BL_096B0BDA8	C2H2	Traes_6AS_FBB2AFAAB
Traes_5BL_096B0BDA8.2	Traes_5BL_096B0BDA8	C2H2	Traes_7AL_96766587F
Traes_5BL_096B0BDA8.2	Traes_5BL_096B0BDA8	C2H2	Traes_5BL_F611D65E0
Traes_5BL_096B0BDA8.2	Traes_5BL_096B0BDA8	C2H2	Traes_3AS_F27BB108C
Traes_5BL_632EBAD09.1	Traes_5BL_632EBAD09	MYB	Traes_2AL_3F3117458
Traes_5BL_7F56360BF.1	Traes_5BL_7F56360BF	C2H2	Traes_4DL_2E9CE89D9
Traes_5BL_7F56360BF.1	Traes_5BL_7F56360BF	C2H2	Traes_1AL_E7144546E
Traes_5BL_9627436AE.1	Traes_5BL_9627436AE	MIKC_MADS	Traes_2DL_E96DCDCB4
Traes_5BL_9627436AE.1	Traes_5BL_9627436AE	MIKC_MADS	Traes_6BL_9CFA54D4A
Traes_5BL_B6051DD37.2	Traes_5BL_B6051DD37	EIL	Traes_2DL_E96DCDCB4
Traes_5BL_F258582BB.1	Traes_5BL_F258582BB	GATA	Traes_4DL_A54C80661
Traes_5BL_F5D379AFC.1	Traes_5BL_F5D379AFC	ERF	Traes_1AL_E7144546E
Traes_5BL_F5D379AFC.1	Traes_5BL_F5D379AFC	ERF	Traes_1DL_C646B6990
Traes_5BL_F5D379AFC.1	Traes_5BL_F5D379AFC	ERF	Traes_2AL_3F3117458
Traes_5BL_F5D379AFC.1	Traes_5BL_F5D379AFC	ERF	Traes_2AL_DFE4C65F6
Traes_5BL_F5D379AFC.1	Traes_5BL_F5D379AFC	ERF	Traes_4BL_B3A1B8342
Traes_5BL_FBC7AF288.1	Traes_5BL_FBC7AF288	ERF	Traes_4BL_B3A1B8342
Traes_5BL_FBC7AF288.1	Traes_5BL_FBC7AF288	ERF	Traes_2AL_3F3117458
Traes_5BL_FBC7AF288.1	Traes_5BL_FBC7AF288	ERF	Traes_2AL_DFE4C65F6
Traes_5BL_FBC7AF288.1	Traes_5BL_FBC7AF288	ERF	Traes_1BL_7C037D478
Traes_5BL_FBC7AF288.1	Traes_5BL_FBC7AF288	ERF	Traes_4DS_88D2821C6
Traes_5BL_FBC7AF288.1	Traes_5BL_FBC7AF288	ERF	Traes_4AS_8D6311711
Traes_5BL_FBC7AF288.1	Traes_5BL_FBC7AF288	ERF	Traes_1AL_E7144546E
Traes_6AL_8BA1FF8B2.1	Traes_6AL_8BA1FF8B2	NAC	Traes_6AL_317133B3F
Traes_6AS_70AD4B0D5.1	Traes_6AS_70AD4B0D5	C3H	Traes_7BL_8CEC8F99B

Traes_6AS_70AD4B0D5.1	Traes_6AS_70AD4B0D5	C3H	Traes_4DL_2E9CE89D9
Traes_6DL_136DE13FB.1	Traes_6DL_136DE13FB	TCP	Traes_5BL_F611D65E0
Traes_6DL_3019F176A.1	Traes_6DL_3019F176A	ERF	Traes_1AL_E7144546E
Traes_6DL_991931CF9.2	Traes_6DL_991931CF9	MYB	Traes_6AL_13BC97E04
Traes_6DL_DD82BD107.2	Traes_6DL_DD82BD107	ERF	Traes_1AL_E7144546E
Traes_6DL_DD82BD107.2	Traes_6DL_DD82BD107	ERF	Traes_2AL_3F3117458
Traes_6DL_DD82BD107.2	Traes_6DL_DD82BD107	ERF	Traes_4AS_8D6311711
Traes_6DL_DD82BD107.2	Traes_6DL_DD82BD107	ERF	Traes_4BL_B3A1B8342
Traes_6DL_DD82BD107.2	Traes_6DL_DD82BD107	ERF	Traes_2AL_DFE4C65F6
Traes_6DL_DD82BD107.2	Traes_6DL_DD82BD107	ERF	Traes_1BL_7C037D478
Traes_6DL_DD82BD107.2	Traes_6DL_DD82BD107	ERF	Traes_4DL_2E9CE89D9
Traes_6DL_EF6D5D4FA.1	Traes_6DL_EF6D5D4FA	Dof	Traes_5AL_07EFD5712
Traes_6DL_EF6D5D4FA.1	Traes_6DL_EF6D5D4FA	Dof	Traes_1AL_E7144546E
Traes_6DL_EF6D5D4FA.1	Traes_6DL_EF6D5D4FA	Dof	Traes_3DL_F32B49981
Traes_6DL_EF6D5D4FA.1	Traes_6DL_EF6D5D4FA	Dof	Traes_7DS_4D01B6175
Traes_6DS_2B2F9C290.2	Traes_6DS_2B2F9C290	C2H2	Traes_5DL_672EE3605
Traes_6DS_2B2F9C290.2	Traes_6DS_2B2F9C290	C2H2	Traes_5BL_F611D65E0
Traes_6DS_2B2F9C290.2	Traes_6DS_2B2F9C290	C2H2	Traes_6DL_4B89E8742
Traes_6DS_2B2F9C290.2	Traes_6DS_2B2F9C290	C2H2	Traes_1AL_095416BC0
Traes_6DS_2B2F9C290.2	Traes_6DS_2B2F9C290	C2H2	Traes_6AL_13BC97E04
Traes_6DS_2B2F9C290.2	Traes_6DS_2B2F9C290	C2H2	Traes_5AL_72A7552B9
Traes_6DS_2B2F9C290.2	Traes_6DS_2B2F9C290	C2H2	Traes_2AL_3F3117458
Traes_6DS_2B2F9C290.2	Traes_6DS_2B2F9C290	C2H2	Traes_1AL_E7144546E
Traes_6DS_2B2F9C290.2	Traes_6DS_2B2F9C290	C2H2	Traes_3DS_57EA31670
Traes_7AL_EA6F4FFDE.2	Traes_7AL_EA6F4FFDE	GATA	Traes_2DS_4CC8FD7E3
Traes_7AL_EA6F4FFDE.2	Traes_7AL_EA6F4FFDE	GATA	Traes_1AL_095416BC0
Traes_7AS_0F019FB93.2	Traes_7AS_0F019FB93	SBP	Traes_6BL_0A9D15EDC
Traes_7DS_433704E8E.1	Traes_7DS_433704E8E	ERF	Traes_2AL_3F3117458
Traes_7DS_433704E8E.1	Traes_7DS_433704E8E	ERF	Traes_2AL_DFE4C65F6
Traes_7DS_433704E8E.1	Traes_7DS_433704E8E	ERF	Traes_4BL_B3A1B8342
Traes_7DS_433704E8E.1	Traes_7DS_433704E8E	ERF	Traes_2BL_7713B3533
Traes_7DS_433704E8E.1	Traes_7DS_433704E8E	ERF	Traes_4AS_8D6311711
Traes_7DS_433704E8E.1	Traes_7DS_433704E8E	ERF	Traes_6AL_13BC97E04
Traes_7DS_433704E8E.1	Traes_7DS_433704E8E	ERF	Traes_6DS_9DD64BD48
Traes_7DS_433704E8E.1	Traes_7DS_433704E8E	ERF	Traes_1BL_7C037D478
Traes_7DS_433704E8E.1	Traes_7DS_433704E8E	ERF	Traes_1AL_E7144546E
Traes_7DS_433704E8E.1	Traes_7DS_433704E8E	ERF	Traes_7AL_1BAB53DCE
Traes_7DS_433704E8E1.1	Traes_7DS_433704E8E1	ERF	Traes_6AL_13BC97E04
Traes_7DS_433704E8E1.1	Traes_7DS_433704E8E1	ERF	Traes_2AL_DFE4C65F6
Traes_7DS_433704E8E1.1	Traes_7DS_433704E8E1	ERF	Traes_2BL_7713B3533
Traes_7DS_433704E8E1.1	Traes_7DS_433704E8E1	ERF	Traes_4BL_B3A1B8342
Traes_7DS_433704E8E1.1	Traes_7DS_433704E8E1	ERF	Traes_1AL_E7144546E
Traes_7DS_433704E8E1.1	Traes_7DS_433704E8E1	ERF	Traes_4AS_8D6311711
Traes_7DS_433704E8E1.1	Traes_7DS_433704E8E1	ERF	Traes_1BL_7C037D478
Traes_7DS_433704E8E1.1	Traes_7DS_433704E8E1	ERF	Traes_6DS_9DD64BD48
Traes_7DS_433704E8E1.1	Traes_7DS_433704E8E1	ERF	Traes_7AL_1BAB53DCE
Traes_7DS_433704E8E1.1	Traes_7DS_433704E8E1	ERF	Traes_2AL_3F3117458

Traes_7DS_FE8BC1125.1	Traes_7DS_FE8BC1125	BBR-BPC	Traes_7AL_D88450A3C
