# Supplementary material for: Comprehensive In Silico Analysis of RNA Silencing-Related Genes and Their Regulatory Elements in Wheat (Triticum aestivum L.)
Source: Biomed Res Int. 2022 Sep 19;2022:4955209. doi: 10.1155/2022/4955209 (PMC9513535; doi:10.1155/2022/4955209)
Supplement: Supplementary 2 — Data S1: protein sequences of the identified DCL genes in wheat. Data S2: protein sequences of the identified AGO genes in wheat. Data S3: protein sequences of the identified RDR genes in wheat. Data S4: list of transcript factors and their families regulating the predicted RNAi-based genes. Data S5: list of cis-regulatory elements associated with the TaDCL protein families. Data S6: list of cis-regulatory elements associated with the TaAGO protein families. Data S7: list of cis-regulatory elements associated with the TaRDR protein families. [file 4955209.f2.zip › Data S5-TaDCL-CRE.pdf]

Data S5: List of *cis*-regulatory elements of DCL genes in wheat(*T.aestivum*)

Functions	Categoires	motifs
cis-acting regulatory element essential for the anaerobic induction	stress/water_log	ARE
core promoter element around -30 of transcription start	other	TATA-box
cis-acting element involved in salicylic acid responsiveness	hormone	TCA-element
cis-acting regulatory element involved in auxin responsiveness	hormone	AuxRR-core
unknown	unknown	MYC
light responsive element	light	3-AF1 binding site
part of a light responsive element	light	Gap-box
unknown	unknown	W box
part of a conserved DNA module involved in light responsiveness	light	Box 4
cis-acting regulatory element	other	Box II -like sequence
unknown	unknown	Myb-binding site
unknown	unknown	AP-1
unknown	unknown	Myc
cis-acting regulatory element involved in the MeJA-responsiveness	hormone	CGTCA-motif
unknown	unknown	MYB recognition site
unknown	unknown	WRE3
cis-acting element involved in defense and stress responsiveness	defense/stress	TC-rich repeats
enhancer-like element involved in anoxic specific inducibility	stress	GC-motif
unknown	unknown	STRE
unknown	unknown	as-1
unknown	unknown	CTAG-motif
cis-acting regulatory element involved in light responsiveness	light	G-Box
part of a conserved DNA module involved in light responsiveness	light	ATC-motif
part of a light responsive element	light	chs-CMA2a
part of a light responsive element	light	TCT-motif
unknown	unknown	MYB
unknown	unknown	Myb
gibberellin-responsive element	hormone	P-box
unknown	unknown	AAGAA-motif
unknown	unknown	CAAT-box
common cis-acting element in promoter and enhancer regions	other	CAAT-box

unknown	unknown	MYB-like sequence
unknown	wound response	WUN-motif
unknown	unknown	ERE
light responsive element	light	Sp1
unknown	unknown	CCGTCC-box
cis-acting regulatory element involved in circadian control	other	circadian
cis-acting regulatory element involved in the MeJA-responsiveness	hormone	TGACG-motif
unknown	unknown	ABRE4
part of a light responsive element	light	I-box
MYBHv1 binding site	other	CCAAT-box
part of a light responsive element	light	GATA-motif
part of a conserved DNA module involved in light responsiveness	light	ATCT-motif
part of a light responsive element	light	GTGGC-motif
unknown	unknown	plant_AP-2-like
cis-regulatory element involved in endosperm expression	hormone	GCN4_motif
cis-acting element involved in low-temperature responsiveness	temperature/stress	LTR
unknown	unknown	CARE
gibberellin-responsive element	hormone	GARE-motif
cis-acting regulatory element	other	A-box
cis-acting regulatory element involved in zein metabolism regulation	hormone	O2-site
light responsive element	light	GT1-motif
auxin-responsive element	hormone	TGA-element
MYB binding site involved in light responsiveness	light	MRE
element involved in differentiation of the palisade mesophyll cells	other	HD-Zip 1
MYB binding site involved in drought-inducibility	drought/stress	MBS
part of a module for light response	light	AE-box
cis-acting element involved in the abscisic acid responsiveness	hormone	ABRE
unknown	unknown	TATA
cis-acting element involved in gibberellin-responsiveness	hormone	TATC-box
part of a light responsive element	light	TCCC-motif
unknown	unknown	ABRE3a
unknown	unknown	CCGTCC motif
unknown	unknown	AT~TATA-box

unknown	unknown	TCA
cis-acting regulatory element related to meristem expression	hormone	CAT-box
protein binding site	other	HD-Zip 3
cis-acting element involved in light responsiveness	light	ACE
MYB binding site involved in flavonoid biosynthetic genes regulation	hormone	MBSI
unknown	unknown	box S
unknown	unknown	ABRE2
unknown	unknown	H-box
part of a light responsive element	light	L-box
unknown	unknown	DRE core
part of a light responsive element	light	Box II
part of a light responsive element	light	GA-motif
unknown	unknown	F-box
element for maximal elicitor-mediated activation (2copies)	other	AT-rich sequence
unknown	unknown	AC-I
unknown	unknown	re2f-1
part of a light responsive element	light	GATT-motif
part of a light responsive element	light	chs-CMA1a
part of a light responsive element	light	LAMP-element
light responsive element	light	4cl-CMA2b
part of a conserved DNA module array (CMA3)	other	3-AF3 binding site
