# Supplementary material for: Comprehensive In Silico Analysis of RNA Silencing-Related Genes and Their Regulatory Elements in Wheat (Triticum aestivum L.)
Source: Biomed Res Int. 2022 Sep 19;2022:4955209. doi: 10.1155/2022/4955209 (PMC9513535; doi:10.1155/2022/4955209)
Supplement: Supplementary 2 — Data S1: protein sequences of the identified DCL genes in wheat. Data S2: protein sequences of the identified AGO genes in wheat. Data S3: protein sequences of the identified RDR genes in wheat. Data S4: list of transcript factors and their families regulating the predicted RNAi-based genes. Data S5: list of cis-regulatory elements associated with the TaDCL protein families. Data S6: list of cis-regulatory elements associated with the TaAGO protein families. Data S7: list of cis-regulatory elements associated with the TaRDR protein families. [file 4955209.f2.zip › Data S6-TaAGO-CRE.pdf]

Data S6: List of *cis*-regulatory elements of AGO genes in wheat(*T.aestivum*)

Functions	Categoires	motifs
unknown	other	W box
wound-responsive element	stress	WUN-motif
part of a light responsive element	light	GA-motif
cis-acting regulatory element involved in circadian control	other	circadian
cis-acting element involved in low-temperature responsiveness	stress	LTR
part of a module for light response	light	AE-box
light responsive element	light	Sp1
enhancer-like element involved in anoxic specific inducibility	hormone	GC-motif
auxin-responsive element	hormone	TGA-element
part of a light responsive element	light	I-box
unknown	other	ERE
cis-acting regulatory element essential for the anaerobic induction	stress	ARE
unknown	other	MYB
unknown	other	as-1
unknown	other	STRE
unknown	other	MYC
cis-acting regulatory element involved in the MeJA-responsiveness	hormone	CGTCA-motif
cis-acting regulatory element involved in auxin responsiveness	hormone	AuxRR-core
unknown	other	TCA
unknown	other	W box
part of a conserved DNA module involved in light responsiveness	light	Box 4
cis-acting regulatory element related to meristem expression	hormone	CAT-box
part of a light responsive element	light	Gap-box
unknown	other	ABRE3a
MYB binding site involved in drought-inducibility	stress	MBS
common cis-acting element in promoter and enhancer regions	other	CAAT-box
light responsive element	light	GT1-motif
unknown	other	WRE3
gibberellin-responsive element	hormone	P-box
unknown	other	MYB-like sequence
part of a light responsive element	light	LAMP-element

core promoter element around -30 of transcription start	other	TATA-box
part of a light responsive element	light	TCCC-motif
cis-acting element involved in the abscisic acid responsiveness	hormone	ABRE
unknown	other	CCGTCC-box
unknown	other	CCGTCC motif
cis-acting element involved in defense and stress responsiveness	stress	TC-rich repeats
cis-acting regulatory element involved in light responsiveness	light	G-box
unknown	other	ABRE4
unknown	other	Myb-binding site
part of a light responsive element	light	TCT-motif
part of a light responsive element	light	GATA-motif
unknown	other	AT~TATA-box
unknown	other	Myc
cis-acting regulatory element involved in the MeJA-responsiveness	hormone	TGACG-motif
unknown	other	AAGAA-motif
cis-acting element involved in salicylic acid responsiveness	hormone	TCA-element
unknown	other	Myb
cis-acting regulatory element	other	A-box
part of a light responsive element	light	chs-Unit 1 m1
unknown	other	MYB recognition site
gibberellin-responsive element	hormone	GARE-motif
cis-acting element involved in light responsiveness	light	ACE
unknown	other	TATA
cis-acting regulatory element involved in seed-specific regulation	hormone	RY-element
part of a light responsive element	light	Box II
cis-acting regulatory element involved in zein metabolism regulation	hormone	O2-site
MYBHv1 binding site	other	CCAAT-box
light responsive element	light	GT1-motif
part of a light responsive element	light	Pc-CMA2c
light responsive element	light	3-AF1 binding site
unknown	other	DRE core
part of a light responsive element	light	L-box
protein binding site	other	Box III

MYB binding site involved in light responsiveness	light	MRE
binding site of AT-rich DNA binding protein (ATBP-1)	other	AT-rich element
part of a light responsive element	light	chs-CMA1a
cis-regulatory element involved in endosperm expression	hormone	GCN4_motif
part of a light responsive element	light	GATT-motif
cis-acting regulatory element involved in seed-specific regulation	hormone	RY-element
unknown	other	AP-1
unknown	other	JERE
unknown	other	CTAG-motif
MYB binding site involved in drought-inducibility	stress	MBS
part of a light responsive element	light	TCCC-motif
cis-acting regulatory element root specific	other	motif I
other	other	HD-Zip 1
unknown	other	box S
unknown	other	CARE
unknown	other	E2Fb
part of a light responsive element	light	chs-CMA2a
unknown	other	AC-I
unknown	other	F-box
cis-acting element involved in gibberellin-responsiveness	hormone	TATC-box
part of a light response element	light	CAG-motif
element involved in differentiation of the palisade mesophyll cells	other	CAAT(A/T)ATTG
MYB binding site involved in flavonoid biosynthetic genes regulation	hormone	MBSI
light responsive element	light	chs-CMA2b
part of a conserved DNA module involved in light responsiveness	light	ATCT-motif
unknown	other	CAAAT
unknown	other	Y-box
part of a conserved DNA module involved in light responsiveness	light	ATC-motif
unknown	other	GC-motif
unknown	other	GRA
unknown	other	dOCT
unknown	other	Box II -like sequence
part of an auxin-responsive element	hormone	AuxRE

unknown	other	DRE1
light responsive element	light	sbp-CMA1c
other	other	3-AF3 binding site
other	other	ACTCATCCT sequence
unknown	other	re2f-1
unknown	other	AT~ABRE
cis-acting element involved in cell cycle regulation	other	MSA-like
part of gapA in (gapA-CMA1) involved with light responsiveness	light	ACA-motif
other	other	CCCCGG
cis-acting regulatory element related to meristem specific activation	other	NON-box
light responsive element	light	4cl-CMA2b
part of a light responsive element	light	GTGGC-motif
unknown	other	AT~ABRE
