# Supplementary material for: Comprehensive In Silico Analysis of RNA Silencing-Related Genes and Their Regulatory Elements in Wheat (Triticum aestivum L.)
Source: Biomed Res Int. 2022 Sep 19;2022:4955209. doi: 10.1155/2022/4955209 (PMC9513535; doi:10.1155/2022/4955209)
Supplement: Supplementary 2 — Data S1: protein sequences of the identified DCL genes in wheat. Data S2: protein sequences of the identified AGO genes in wheat. Data S3: protein sequences of the identified RDR genes in wheat. Data S4: list of transcript factors and their families regulating the predicted RNAi-based genes. Data S5: list of cis-regulatory elements associated with the TaDCL protein families. Data S6: list of cis-regulatory elements associated with the TaAGO protein families. Data S7: list of cis-regulatory elements associated with the TaRDR protein families. [file 4955209.f2.zip › Data S7-TaRDR-CRE.pdf]

Data S7: List of *cis*-regulatory elements of RDR genes in wheat(*T.aestivum*)

Functions	Categoires	motifs
unknown	other	as-1
unknown	other	W box
unknown	other	CCGTCC motif
unknown	other	AT~TATA-box
cis-acting element involved in the abscisic acid responsiveness	hormone	ABRE
part of a light responsive element	light	chs-CMA1a
unknown	other	CAAT-box
unknown	other	ABRE3a
unknown	other	MYB
part of a light responsive element	light	GATA-motif
part of a light responsive element	light	GA-motif
cis-acting element involved in gibberellin-responsiveness	hormone	TATC-box
cis-acting regulatory element essential for the anaerobic induction	stress	ARE
unknown	other	JERE
unknown	other	MYB recognition site
unknown	other	TATA-box
unknown	other	CTAG-motif
gibberellin-responsive element	hormone	GARE-motif
part of a light responsive element	light	TCCC-motif
unknown	other	TCA
auxin-responsive element	hormone	TGA-element
cis-acting element involved in salicylic acid responsiveness	hormone	TCA-element
MYBHv1 binding site	other	CCAAT-box
unknown	other	box S
unknown	other	ERE
cis-acting regulatory element related to meristem expression	hormone	CAT-box
unknown	other	MYC
part of a conserved DNA module involved in light responsiveness	light	CARE
part of a conserved DNA module involved in light responsiveness	light	Box 4
cis-acting regulatory element	other	A-box
unknown	other	AAGAA-motif

unknown	other	Myb
cis-acting regulatory element involved in circadian control	other	circadian
cis-acting regulatory element involved in the MeJA-responsiveness	hormone	TGACG-motif
gibberellin-responsive element	hormone	P-box
MYB binding site involved in drought-inducibility	stress	MBS
unknown	other	WUN-motif
unknown	other	Myb-binding site
cis-acting regulatory element involved in zein metabolism regulation	hormone	O2-site
unknown	other	CCGTCC-box
unknown	other	WRE3
cis-acting regulatory element involved in light responsiveness	light	G-box
unknown	other	DRE core
unknown	other	ABRE4
unknown	other	STRE
part of a conserved DNA module involved in light responsiveness	light	ATC-motif
unknown	other	MYB-like sequence
cis-acting regulatory element involved in light responsiveness	light	G-Box
cis-acting element involved in defense and stress responsiveness	stress	TC-rich repeats
light responsive element	light	GT1-motif
cis-acting regulatory element involved in the MeJA-responsiveness	hormone	CGTCA-motif
part of a light responsive element	light	LS7
cis-acting regulatory element involved in seed-specific regulation	hormone	RY-element
part of a light responsive element	light	TCT-motif
part of a light responsive element	light	chs-CMA2a
part of a module for light response	light	AE-box
unknown	other	Myc
unknown	other	Box II -like sequence
binding site of AT-rich DNA binding protein (ATBP-1)	other	AT-rich element
cis-acting element involved in low-temperature responsiveness	stress	LTR
part of a light responsive element	light	LAMP-element
cis-acting element involved in cell cycle regulation	other	MSA-like
unknown	other	AC-I
light responsive element	light	Sp1

enhancer-like element involved in anoxic specific inducibility	hormone	GC-motif
unknown	other	DRE1
cis-regulatory element involved in endosperm expression	hormone	GCN4_motif
part of a conserved DNA module involved in light responsiveness	light	ATCT-motif
unknown	other	dOCT
unknown	other	OCT
unknown	other	AT~ABRE
protein binding site	other	HD-Zip 3
MYB binding site involved in light responsiveness	light	MRE
part of a light responsive element	light	sbp-CMA1c
part of a light responsive element	light	GTGGC-motif
cis-acting regulatory element involved in auxin responsiveness	hormone	AuxRR-core
unknown	other	Box III
cis-acting element involved in light responsiveness	light	ACE
part of a light responsive element	light	I-box
part of a light responsive element	light	chs-Unit 1 m1
part of a light responsive element	light	Gap-box
part of an auxin-responsive element	hormone	TGA-box
unknown	other	TATA
light responsive element	light	3-AF1 binding site
unknown	other	NON
element involved in differentiation of the palisade mesophyll cells	hormone	HD-Zip 1
unknown	other	re2f-1
part of a light responsive element	light	L-box
unknown	other	H-box
unknown	other	AP-1
part of a conserved DNA module array (CMA3)	other	3-AF3 binding site
element for maximal elicitor-mediated activation (2copies)	other	AT-rich sequence
