# Supplementary material for: Comprehensive In Silico Analysis of RNA Silencing-Related Genes and Their Regulatory Elements in Wheat (Triticum aestivum L.)
Source: Biomed Res Int. 2022 Sep 19;2022:4955209. doi: 10.1155/2022/4955209 (PMC9513535; doi:10.1155/2022/4955209)
Supplement: Supplementary 3 — Figure S1: alignment of catalytic regions in RdRp domains of RDR proteins of wheat, rice, and A. thaliana. Figure S2: discovery of 20 conserved motif structures. Figure S3A-C: three-dimensional protein structure of the (A) TaDCL, (B) TaAGO, and (C) TaRDR proteins of wheat. Figure S4: regulatory gene network among the TF families and the predicted RNAi-based genes in wheat. Figure S5: distribution of TF families corresponding to each RNAi gene member. Figure S6: RNAi gene-mediated subnetworks with different TF families. (A) ERF, (B) MIKC-MADS, (C) C2H2, (D) BBR-BPC, (E) MYB, and (F) Dof TF family. [file 4955209.f3.zip › Figure S1.pdf]

```
               *       880         *       900         *       920         *       940         *       960         *
AtRDR1   : PVVVAKNPCLHPGDVRVLQAVNVP--ALNHMVDC---VVFPQKGLRPHPNECSGSDLDGDIYFVCWDQELVP----------PRTSEPMDYTPEPTQILDHDVTIEEV :  839
AtRDR2   : KVVVTKNPCLHPGDIRVLDAIYEVHFEEKGYLDC---IIFPQKGERPHPNECSGGDLDGDQFFVSWDEKIIP----------SEMDPPMDYAGSRPRLMDHDVTLEEI :  872
AtRDR3   : DVLVYRNPGLHFGDIHVLKATYVKALED-YVGNAKFAVFFPQKGPRSLGDEIAGGDFDGDMYFISRNPKLLEHFKPSEPWVS-SSKPSKIYCGRKPSELSEEELEEEL :  744
AtRDR4   : DVLVYRNPGLHFGDIHILKATYVKSLEQ-YVGNSKYGVFFPQKGPRSLGDEIAGGDFDGDMYFISRNPKLLEHYKPSEPWVS-SSPRSKIYTGRQPSELSPEQLEEEL :  733
AtRDR5   : EVLVYRNPGLHFGDIHILKATYVKALEE-YVGNSKFAVFFPQKGPRSLGDEIAGGDFDGDMYFISRNPELLENFKPSEPWVS-LTPPSKSNSGRAPSQLSPEELEEEL :  733
AtRDR6   : YVAIAKNPCLHPGDVRILEAVDVP--QLHHMYDC---LIFPQKGDRPHTNEASGSDLDGDLYFVAWDQKLIPPN--------RKSYPAMHYDAAEEKSLGRAVNHQDI :  907
OsSHL2   : TVVIAKNPCLHPGDVRILEAVDVP--ELHHLVDC---LVFPQKGERPHANEASGSDLDGDLYFVTWDEKLIPPG--------KKSWNPMDYSPPEAKQLPRQVSQHDI :  931
OsRDR1   : KVVIAKNPCLHPGDIRILHAVDVP--VLHHMFNC---VVFPQQGPRPHPNECSGSDLDGDIYFVSWDPSLIP----------PRMVTPMDYTPAPTETLDHDVTIEEV :  471
OsRDR2   : KVAITKNPCLHPGDIRVLEAIYDPDLVG--MVDC---LVFPQRGERPHPNECSGGDLDGDLYFITWDDKLIP----------EKVDTPMDYTATRPRIMDHVVTLEEI :  866
OsRDR3   : DVLVYKHPGLHFGDIHVLKATYIRDLEKEYVGYAKYAILFPISGPRSLADEMANSDFDGDIYWVSKNPKLLEHFKPSEPWV--QAIKPKKTKQKKPQDCNESKLERLL :  181
OsRDR4   : DVLVYKYPGLHPGDIHVLKATYSSDIEK-VVGNSKHAILFPTTGQRSLADEMANSDFDGDIYWVSLNPKLLEHFKPSKPWV--PAITPNGTKQKGPEDFNESELERVL :  326
TaRDR2a  : KVAVSKNPCLHPGDIRVLEAVYDHGLYAKNLVDC---VVFPQRGERPHPNECSGGDLDGDLYFITWDEKLIP----------EEVDSPMDYTAARPRIMDHVVTLEEI :  686
TaRDR5   : NVLVYKHPGLHFGDIHVLTSRYIEDIHD-VVGYSRYAILFPTSGPRSLADEMANSDFDGDMYWVSINEQLLKQFKPSKPWEWGQVNKPIQAEKKCLLDLDEPLLDRSL :  450
TaRDR2b  : RVAVSKNPCLHPGDIRVLEAVYDHGLYAKNLVDC---VVFPQRGERPHPNECSGGDLDGDLYFITWDEKLIP----------EKVDSPMDYTAARPRIMDHVVTLEEI :  870
TaRDR1d  : KVVVAKNPCIHPGDIRILQAVHSP--PLGHMVNC---VVFPQLGPRPHPNECSGSDLDGDIYFVSWDPDLIP----------TRMVAPMDYTPAPTETLDHDVMIEVF :  478
TaRDR1a  : KVVVAKNPCLHPGDIRILEAVYTP--VLDHMVNC---VVFPQQGPRPHPNECSGSDLDGDIYFVSWDPDLIP----------TRMVAPMDYTPAPTETLDHDVMIEEV :  845
TaRDR6a  : TVVMAKNPCLHPGDVRILEAIDVP--ALHHLVDC---LVFPKNGERPHANEASGSDLDGDLYFVTWDEKLIPPG--------KRSWNPMDYSPAEAKQLPRKVTQSDI :  637
TaRDR2d  : KVAVSKNPCLHPGDIRVLEAVYDHGLYAKNLVDC---VVFPQRGERPHPNECSGGDLDGDLYFITWDEKLIP----------EKVDSPMDYTAARPRIMDHVVTLE-- :  869
TaRDR1e  : ------------------------------MVNC---VVFPQLGPRPHPNECSGSDLDGDIYFVSWDPDLIP----------TRMVAPMDYTPAPTETLDHDVMIEEV :   65
TaRDR6b  : TVVMAKNPCLHPGDVRILEAIDVP--ALHHLVDC---LVFPKNGERPHANEASGSDLDGDLYFVTWDEKLIPPG--------KRSWNPMDYSPAEAKQLPRKVTQSDI :  460
TaRDR1f  : KVVVAKNPCIHPGDIRILQAVQSP--LLGHMVNC---VVFPQLGPRPHPNECSGSDLDGDIYFVSWDPDLIP----------TRMVAPMDYTPAPTETLDHDVMIEEV :  844
TaRDR1b  : KVIVAKNPCLHPGDIRILEAVYTP--VLDHMVNC---VVFPQQGPRPHPNECSGSDLDGDIYFVSWDPDLIP----------TRMVAPMDYTPAPTETLDHDVMIEEV :  462
TaRDR3   : EVLVYRHPGLHFGDIHVLTATYSEAIQD-FVGDSKYAILFPVSGPRSLADEMAGGDFDGDMYWVSRNPQLLKYFKPSEPWD--PRNPPRKAKQEKPQDYDESKLEHIL :  403
TaRDR4   : EVLVYRHPGLHFGDIHVLTATYSEAIQD-FVGDSKYAILFPVSGPRSLADEMAGGDFDGDMYWVSRNPQLLKYFKPSEPWD--PRSPPRKAKQEKPQDYDESKLEHIL :  181
TaRDR2c  : KVAISKNPCLHPGDIRVLEAVYDHGLYANNLVDC---VVFPQRGERPHPNECSGGDLDGDLYFITWDEKLIP----------EKVDSPMDYTAARPRIMDHVVTLEEI :  866
TaRDR1c  : KVVVAKNPCLHPGDIRILEAVYTP--VLDHMVNC---VVFPQQGPRPHPDECSGSDLDGDIYFVSWDPDLIP----------TRMVAPMDYTPAPTETLDHDVMIEEV :  844
TaRDR1g  : KVVVAKNPCIHPGDIRILQAVHSP--PLGHMVNC---VVFPQLGPRPHPNECSGSDLDGDIYFVSWDPDLIP----------TRMVAPMDYTPAPTETLDHDVMIEEV :  675
            v     p  h gd   l a                 6 FP  G R   1E  g D DGD 556  1  66                p
```
